# Does task experience moderate habituation effects in drop jumps? - An intra- and interday reliability and measurement error analysis

**DOI:** 10.1186/s13102-026-01833-3

**Published:** 2026-06-29

**Authors:** Konstantin Warneke, Lukas Guschel, Nina Claassen, Stanislav D. Siegel, Michael Keiner

**Affiliations:** 1 Institute of Sustainability Psychology (ISP), Leuphana University Lüneburg, Lüneburg, Germany; 2 Institute of Sport Science, Department for Human Movement Science and Exercise Physiology, Friedrich Schiller University Jena, Seidelstraße 20, 07747 Jena, Germany; 3 Institute of Human Movement Science and Exercise Physiology, University Osnabrück, Osnabrück, Germany; 4 Experimental Orthopaedics, University Hospital Jena, Campus Eisenberg, Waldkliniken Eisenberg, Friedrich-Schiller-Universität Jena, Jena, Germany; 5 Department for Sport Science, German University of Health and Sport, Ismaning, Germany

**Keywords:** Reactive Strength Index, Repeatability, Test consistency, Task familiarization, Practice effects

## Abstract

**Background:**

Drop jumps (DJs) are common tests for determining stretch-shortening cycle efficiency. Despite their wide implementation, numerous studies did not adhere to validity guidelines, and participants reached ground contact times (GCTs) of > 250ms. The DJ is a coordinatively-demanding task and it could be hypothesized that a lack of adherence to GCT thresholds could be attributed to insufficient test familiarization. Accordingly, this study evaluated the impact of extensive task habituation on test-retest reliability and GCT adherence.

**Methods:**

Eighty-nine healthy participants were allocated to an experienced and inexperienced subgroup. All subjects performed DJs on five days from three different drop heights, with three trials per height (5 × 3 × 3 DJs = 45 jumps). Reliability coefficients and test-retest systematic and random errors were quantified between testing days.

**Results:**

The inexperienced subgroup showed systematic performance increases between test day 1 and the subsequent days in most evaluated parameters with largest effects obtained for GCTs (up to d = 0.83, *p* < 0.001). With up to 30%, the MAPEs peaked for reactive strength index (RSI) in the day 1 to day 5 comparison. No systematic test-retest increases occurred in the experienced group, and the random percentage error was meaningfully smaller (16–17% for the RSI).

**Conclusion:**

The systematic bias observed in the inexperienced subgroup might indicate significant task habituation, while the experienced group was already familiar with the testing procedure and showed no test-retest improvements. Since subgroup measurement errors differed meaningfully (e.g. 16% versus 30% RSI MAPE), future DJ research should provide their own reliability quantifications and sufficient habituation.

**Supplementary Information:**

The online version contains supplementary material available at 10.1186/s13102-026-01833-3.

## Introduction

Assessments of strength and power are fundamental for performance and health monitoring, for example in elite sports [1] or in return to sports rehabilitation [2]. Across a variety of testing methods, standardized jump tests are one of the most frequently applied diagnostic methodologies in athletic settings [1, 3, 4]. Beyond the squat jump (SJ) and countermovement jump (CMJ), the drop jump (DJ) is commonly applied to evaluate explosive and speed performance, as it monitors an athlete’s ability to potentiate performance by using the short stretch-shortening cycle (SSC) [5, 6].

To accurately evaluate the SSC, the participant drops from a box of a prescribed height which is commonly between 0.15 and 0.60 m. The ground contact time (GCT) should be kept as brief as possible by explosively plantarflexing the ankle joints (ankle “snapping”), without a heel drop and/or excessive knee flexion [7]. To ensure a valid DJ, a maximum GCT of 250ms was recommended by Schmidtbleicher [6, 8]. In combination with the realized jump height, the GCT is crucial for the calculation of the Reactive Strength Index (RSI), which represents the most common reactive strength performance parameter in the literature [7]. These strict standardizations require athletes to adhere to these quality criteria reliably.

In general, reliability plays a key role in internal data validity [9, 10]. Intraday reliability/repeatability provides the baseline for further interpretations and is of critical importance for internal data validity [11]. Most commonly, reliability is quantified via intraclass correlation coefficients (ICCs) which have demonstrated sufficient reliability in previous studies (ICC = 0.73 to 0.99) [12–18]. Referring to previous ICCs, reliability was indicated to be transferable from study to study without the need to assess reliability metrics in each individual study.

However, reliability and outcome stability depend on the participants’ ability to adhere to the task requirements and thus, on test familiarization and previous experience. In strength testing, Ritti-Dias et al. [19] showed that in one repetition maximum strength tests, participants who were familiar with the measurement protocol were able to repeat the strength test without meaningful systematic test-retest discrepancies, while their inexperienced counterparts showed significant habituation effects reflected in performance improvements after repeated testing. Similar results were reported for young and older adults and were observed in athletic populations for isometric strength tests. Depending on the test and the population included, up to eight familiarization/habituation sessions were necessary to remove significant habituation effects [20–22].

Although numerous studies included untrained or recreationally active participants without the ability to adhere to strict standardizations (e.g. GCT < 250ms) [14–17, 23] numerous studies did not calculate test-retest reliability [24–26] and research focusing on habituation effects in DJ research is scarce.

Accordingly, this study will address the following research questions: (a) Does previous DJ test experience moderate reactive strength performance key parameters? (b) Does task habituation result in shortened GCT? (c) Does task habituation differ between participants with and without previous DJ test experience? Accordingly, the primary hypothesis was that (1) DJ parameters will undergo significant habituation effects (test-retest mean improvements), (2) task habituation will reduce the random error margins, and (3) that such effects will occur primarily in participants without sufficient familiarization with the test.

## Materials and methods

### Study design, conceptual assumptions and evaluation approach

**Fig. 1:**
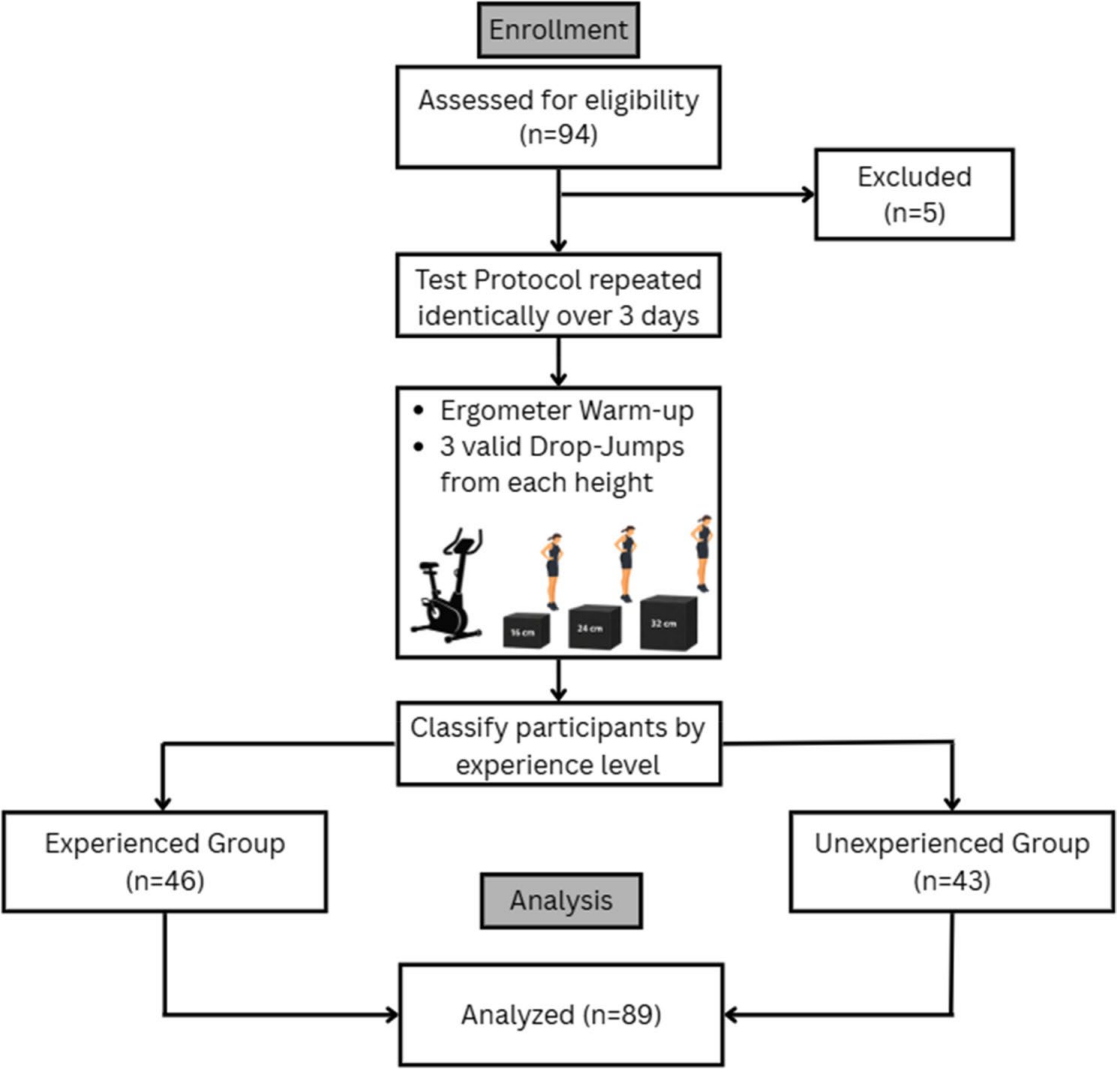
Graphical illustration of the experimental design

Hypotheses were tested over five days in a maximal time window of one week, in which 45 DJ trials (3 trials per day, 3 heights per day, 5 testing days) were conducted by all participants without an intervention between testing days. The testing protocol adheres to current reliability guidelines requesting at least three trials per test [27] and including > 1 drop height [7, 14, 28] (Fig. 1).

### Rationale for measurement error analysis

While the ICC provides relevant information about test-retest reliability and the consistency of the measurement, it does not quantify different sources of measurement errors (e.g. systematic test-retest increases and/or biological variability and other sources of random errors) [10, 29]. In the literature, the additional implementation of Bland Altman analyses with an evaluation of the level of significance (e.g. paired sampled t-test or Analysis of Variance (ANOVA)) [10] were proposed to quantify task habituation. Beyond the systematic error, Hopkins [9] outlined the relevance of random error quantifications to avoid excessive interpretations of data and account for biological variability or unsystematic inter-subject variances in task habituation velocities. These should include the Standard error of measurement (SEM) [30], Typical error [9], or the mean absolute error (MAE), which can be expressed in percent as the Mean Absolute Percentage Error (MAPE) [31].

Since each approach has its benefits and caveats, Atkinson & Nevill [10] recommended the quantification of more than one metric accounting for error sources to enhance interpretability (see section "Drop Jump testing" for calculations).

### Subjects

#### Sample size determination

A G-Power a priori sample size estimation is not applicable for reliability studies due to two main reasons: (1) There is no option to calculate the sample size for reliability metrics in the software and (2) assuming high magnitude effects (e.g. ICCs) would reduce the required sample size, as assuming large sample sizes would require smaller samples to reach adequate statistical power. This stood in direct conflict with current evidence showing that ICCs and random error quantifications fluctuate in small sample sizes.

Based on Koo & Li [27], a minimum sample size of 30 is required to reasonably calculate ICCs. Hopkins [9] described reliability studies with less than 50 participants as pilot data. This participant number is also considered the minimal sample size for valid Bland Altman (BA) analyses [29, 32]. Since small, but common sample sizes showed unstable point estimates (adding or removing only five participants changed the classification of reliability and doubles/halves the MAPE in a boot-strap experiment) a larger required sample size approaching 100–150 participants was proposed, which resulted in more stable MAPEs and reduced 95%CI margins of the ICC [33].

Accordingly, this study sought to enhance the sample size compared to previous DJ reliability research that focused on smaller sample sizes ranging between 12 and 37 participants [13–17, 23]. Only few studies were found including higher sample sizes with *n* > 50, for example, *n* = 79 [12].

#### Participants

Initially, 94 participants were recruited from the sports education program of the local university, but 5 participants had to be removed from the results evaluation as they did not complete all five testing sessions. As a consequence a total sample size of 89 physically active and healthy participants (male: *n* = 46, age: 24.5±5.6 years old, mass: 79.3±6.8 kg, height: 182.3±4.3 cm, female: *n* = 43, age: 23.9±3.7 years old, mass: 63.4±4.8 kg, height: 161.4±4.2 cm) were included to the study.

#### Eligibility criteria

Participants were included if they fulfilled the following requirement(s): physically and recreationally active, which means that they met the World Health Organization minimum activity guidelines (at least 150–300 min of moderate – intensity activities or 75–150 min of vigorous – intensity activity a week). Furthermore, participants should regularly train with strengthening activities twice per week [34]. Participants were excluded if they suffered from injuries that caused immobilization or inactivity within the last six months. Furthermore, orthopedic or neurological patients were not considered.

#### Subgroup allocation (experienced/inexperienced)

To evaluate the influence of learning/habituation effects on DJ internal validity and the ability to adhere with cornerstones of DJ performance (i.e., keeping the GCT≤250ms), participants were divided into two subgroups.

Since no globally accepted guideline on how to explore experience with DJ exists, participants provided information about their previous DJ experience and if they were familiar with the test protocol, as previously performed by Ritti-Dias et al. [19]. If they stated “no”, they were allocated to the inexperienced subgroup.

However, previous experience does not automatically mean that participants were already able to perform DJ appropriately. Since it is possible that participants had sporadic experiences or did not learn how to appropriately perform a valid DJ, these participants were allocated into the inexperienced group. Therefore, if participants could not attain DJ with GCTs < 250ms in the first drop height (16 cm), they were allocated to the inexperienced subgroup.

**Fig. 2:**
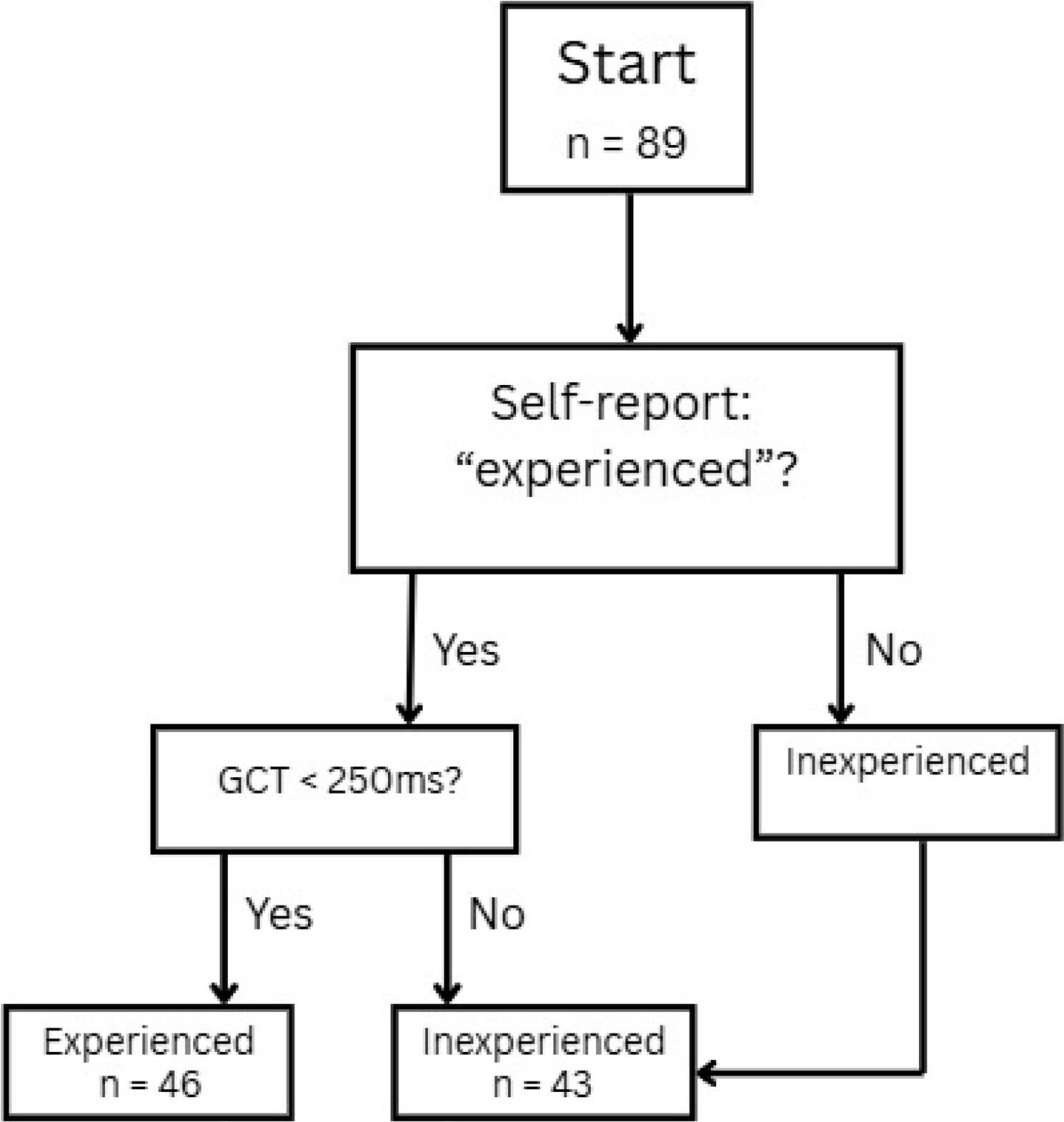
Shows the decision tree for participant allocation into experienced and inexperienced subgroups

Accordingly, only participants fulfilling both criteria (1. experience with DJ and 2. GCTs of < 250ms) were eligible for the experienced group. This procedure resulted in similar group sizes of *n* = 47 (experienced) and *n* = 47 (inexperienced) group. The drop out reduced the subgroup size in the inexperienced group by four and in the experienced group by 1, resulting in *n* = 46 (experienced) and *n* = 43 (inexperienced), see Fig. 2.

The participants were instructed to avoid strenuous physical activity 24 h before testing and were scheduled at the same time of the day for each test session. All participants provided written informed consent. The study was performed in adherence with the Declaration of Helsinki, and the protocol was ethically approved by the local ethical review board (No. FSV 25/096).

### Drop jump testing

The DJ is the most common test to monitor the athlete’s ability to use a rapid SSC for jump performance potentiation. Therefore, athletes must adhere to minimal GCTs with a maximum of 250ms [6] and without excessive knee joint flexion to store and release elastic energy primarily from the Achilles tendon and m. triceps surae. The instructional focus must be on short GCTs while seeking maximal jump heights. Furthermore, whether a participant can adequately use the SSC should be evaluated using more than one jump height [7, 14, 28].

#### Testing procedure

DJs were accordingly performed from three different heights (16 cm, 24 cm and 32 cm) recorded by a force plate (Bertec, USA) with a sampling rate of 2700 Hz. Since previous studies outlined relevance of the verbal instruction for movement execution (e.g. external focus, internal focus, focus on shortest GCTs possible [35–37] participants were instructed to keep GCTs minimal while simultaneously jumping as high as possible.

**Fig. 3:**
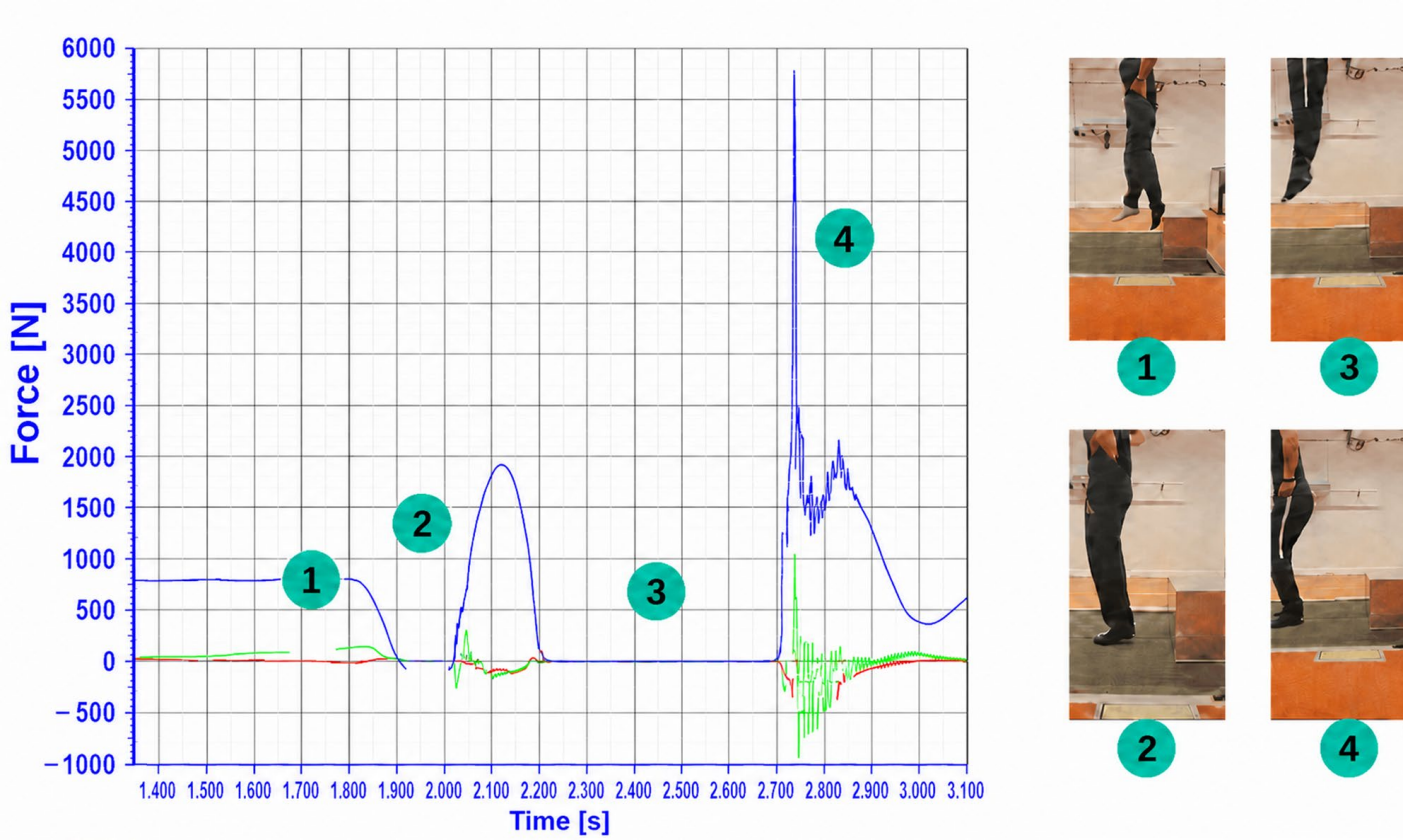
Shows a valid drop jump force-time curve with (1) showing the drop, (2) the landing, (3) the flight time and (4) the landing

To standardize and account for inexperienced participants [36], the instruction advice from Barillas et al. [37] was used and a combined cue (jump as high as possible with shortest GCTs as possible) was adopted. Additionally, extensive knee flexion and heel dropping when landing was avoided. Intentionally, the 250ms GCT threshold was not considered as a critical inclusion criterion in the first session, as the experiment was conducted to evaluate whether habituation improved the outcome. All three drop heights were tested every day over five days in a randomized order. Three trials were performed per height per day. For interday calculations, the mean of the three trials per height were used. The DJ movement as well as a force-time curve are exemplary illustrated in Fig. 3.

### Analysis of force platform data

A custom MATLAB script was used for data analysis. Raw vertical force was low-pass filtered with a fourth-order Butterworth filter (50 Hz). Initial contact and toe-off were defined using a 50 N threshold. GCT was calculated from force onset (> 50 N) to toe-off (< 50 N), and flight time from toe-off to the subsequent initial contact. Jump height was calculated from flight time using the equation: $\:JH=\frac{g\cdot\:{t}_{f}^{2}}{8}$, where $\:JH$= jump height (m), $\:g$= gravitational acceleration (9.81 m·s⁻²), $\:{t}_{f}$= flight time (s). The RSI was calculated as follows: $\:RSI=\frac{JH}{GCT}$.

### Statistical analyses

Statistical analysis was performed via JASP (Version 0.18.3 (Intel), Netherlands). Normal distribution was investigated via the Shapiro-Wilk test. DJ performance parameters were provided as Mean (M) and Standard Deviation (SD) and supplemented by Median and 25% and 75% percentiles if the normal distribution assumption was violated. The reliability analysis was performed separately for intra- and interday test-retest comparisons and stratified by DJ experience level, using reliability and measurement error quantifications as described in section "Study design, conceptual assumptions and evaluation approach" For interday comparisons, the mean of the three intraday tests was used for further data processing. Details were reported on relative and absolute reliability as well as the systematic and random error. In adherence to Koo & Li [27] the two-way intraclass correlation coefficient (ICC) for agreement (3,k) was chosen to estimate reliability with at least three columns (three intraday and five interday trials). The following formula was adopted:

$$\:ICC=\frac{{MS}_{R}-{MS}_{E}}{\frac{({MS}_{R}+\left({MS}_{C}-{MS}_{E}\right))}{n}}$$

Where ICC = intraclass correlation coefficient. $\:{\mathrm{M}\mathrm{S}}_{\mathrm{C}}$ = mean square for columns,$\:\:{\mathrm{M}\mathrm{S}}_{\mathrm{E}}$ = mean square for error, $\:{\mathrm{M}\mathrm{S}}_{\mathrm{R}}$ = mean square for rows, n = Number of subjects.

Reliability was classified in accordance with Koo & Li [27] as poor (ICC < 0.5), moderate (ICC ≥ 0.5 – <0.75), good (ICC ≥ 0.75 – <0.9) and excellent (ICC ≥ 0.9). Using the ICC, the Standard Error of Measurement (SEM) was calculated as follows [29]:

$$\:SEM=SD*\sqrt{1-ICC}$$

Where SEM = standard error of measurement, SD = standard deviation of the mean differences between trial 1 and 2 and ICC = intraclass correlation coefficient.

With the aim to receive information about practical relevance of the SEM regarding longitudinal changes, the minimal detectable change (MDC) was calculated as follows:

$$\:MDC=SEM*1.96*\sqrt{2}$$

Where MDC = minimal detectable change and SEM = standard error of measurement.

The systematic error evaluates test-retest differences via inference statistics using a one-way Analysis of Variance (ANOVA) with post-hoc testing (here: Bonferroni test). Effect sizes were interpreted via partial eta squared (ⴄ_p_²) and classified as follows: ⴄ_p_²<0.06 as small, ⴄ_p_² ≥0.06 – <0.14 as moderate and ⴄ_p_² ≥ 0.14 as large effects. Post-hoc individual effects were classified via Cohen’s d: d < 0.2 as trivial, d ≥ 0.2 – < 0.5 as small, d ≥ 0.5 – < 0.8 as moderate and d ≥ 0.8 as large effect sizes. As non-parametric tests, the Kruskall Wallis test with the Dunn’s post hoc test as well as the Friedman test with the Conover post hoc test were additionally calculated as a confirmation of parametric test results in case of violated normal distribution.

The random error was quantified using the Mean Absolute Error (MAE) which is calculated as follows:

$$\:MAE=\frac{1}{n}*\sum\:_{i=1}^{n}\left|{x}_{i}-{y}_{i}\right|$$

Where *n* = number of data point, *i* = index for each (paired) data point, *x*_*i*_ = *i*-th data point in variable *x* (e.g., jump height in trial one) *and y*_*i*_ = *i*-th data point in variable *y* (e.g., jump height in trial two).

Adopting the adjusted formula from Warneke et al. [31], the Mean Absolute Percentage Error (MAPE) was calculated to express the random error measurement unit-free.

$$\:\mathrm{M}\mathrm{A}\mathrm{P}\mathrm{E}=\frac{1}{n}*\sum\:_{i=1}^{n}\left|\frac{{(x}_{i}-{y}_{i})}{\left(\frac{{x}_{i}+{y}_{i}}{2}\right)}\right|*100$$

Where *n* = number of data point*s*,* i* = index for each (paired) data point, *x*_*i*_ = *i*-th data point in variable *x* (e.g., jump height in trial one) and *y*_*i*_ referred to the *i*-th data point in variable *y* (e.g., jump height in trial two).

Additionally, presuming DJ to be a stable performance parameter measured over time, an agreement analysis was supplemented via Bland Altman analyses [32]. The graphical illustration was performed using RStudio via packages ggplot2 and BlandAltmanLeh. If inference statistics were performed, the α-error was set to 0.05. If multiple tests were performed per hypothesis, the level of significance was adjusted via family-wise error rate (FWER) correction.

## Results

### Assumption evaluation

While for several parameters the normal distribution assumption remained unviolated (e.g. jump height 16 (test 2 on day 1), GCTs 16 day 1, RSI 24 day1), in others, the Shapiro-Wilk test surpassed the *p* = 0.05 threshold (e.g. jump height 32 day 1 all tests, RSI 32 day 1 test 1 and 3). To keep homogeneity in results reporting, due to the large sample size, the ANOVA was still used for systematic error analysis as it was shown to be robust against normal distribution violation with *n* > 30 [38, 39]. Nevertheless, the interpretations were confirmed via non-parametric tests.

### Intraday reliability

#### Reliability coefficients

Intraday reliability coefficients ranged between low to excellent (ICC = 0.54–0.98, lowest 95% CI border at 0.42). Jump height and RSI ICCs were mostly excellent ICC > 0.9. Exceptions were jump height and RSI on day 3 and 4 (e.g. day4 RSI16: ICC = 0.59, 0.48–0.69 95% CI or JH24: ICC = 0.80, 0.73–0.86 95% CI). Worse and less stable intraday reliability was observed for GCTs ranging from poor to excellent (ICC = 0.54–0.87, 0.42–91 95% CI). For more detailed results, see Supplemental Table S1.

Subgroup ICCs indicated good to excellent reliability (ICC lower confidence interval > 0.75) with some test indicating subgroup differences. For instance, on day 1, GCT16 in the inexperienced sub-sample reached an ICC = 0.75, 0.60–0.86, 95%CI while the experienced group showed an ICC = 0.94, 0.89–0.97, 95% CI. In contrast, on test day 4, the experienced subgroup reached an RSI16 ICC of 0.70, 0.50–0.83 95% CI, which was below the reliability of the inexperienced subgroup (ICC = 0.92, 0.88–0.96 95% CI). More detailed results can be reviewed in the Supplemental Table S2).

#### Systematic measurement errors

Across the parameters, there were no significant systematic test-retest changes in the overall population (*p* = 0.294–0.991) (Supplemental Table S1). In the subgroup analysis, there were individual small magnitude effects in JH16 and GCT16 (d = 0.26–0.41, *p* < 0.001–0.03,) in the inexperienced participants. Detailed subgroup results can be reviewed in the Supplemental Table S2.

#### Random measurement errors

In the overall sample, measurement errors (MAPE) across the parameters ranged between 5.32 and 17.69%. Over the testing days, a decreasing tendency of the intraday measurement errors was observed, e.g. RSI16 13.78–17.69% on test day 1 and 10.31–11.99% on day 5. Jumping height MAPEs (5.78–8.35%) were generally smaller compared to GCTs and RSIs. Detailed results for each testing day and trial can be reviewed in the Supplemental Table S1.

There were subgroup differences for several parameters. In the RSI16, the intraday MAPE was 9.74–13.30% in the experienced, while the inexperienced participants reached MAPE = 17.40–21.86%. Similarly, on day one JH16, the experienced subgroup random error was close to half as high (MAPE = 5.47–9.51%) compared to the inexperienced subgroup (13.70–18.18%). Similarly, on day two the GCT24 the experienced subgroup MAPEs were with 6.08–6.98% smaller compared to those observed in the experienced group with 12%. In contrast, with a MAPE of 5.65–8.09%, the experienced group showed slightly higher measurement errors than their inexperienced counterparts (MAPE = 7.29–7.76%) in JH32 on day one. Further details and all remaining subgroup differences can be reviewed in the Supplemental Table S2.

### Interday reliability

#### Interday reliability coefficients

In the overall sample, ICCs were good to excellent with the lowest ICCs observed for GCTs measures. Jump height and RSI indicated excellent reliability with all ICCs and their 95%CIs being > 0.9) (see Table 1).

**Table 1:** Interday reliability metrics for drop jump performance metrics stratified for drop heights accounting for descriptives, relative reliability as well as random and systematic measurement errors in the overall study population

	Parameter	M±SD	Median (25–75% Percentiles	ICC	SEM	MDC	ANOVA	Sig. post-hoc	MAE _MIN−MAX_	MAPE _MIN_MAX_
16 cm	1JH	0.214±0.07	0.21 (0.15–0.26)	0.963 (0.949–0.974)	0.004	0.012	*p* = 0.699	N/A	0.019–0.023	9.21–10.48
	2JH	0.221±0.06	0.22 (0.18–0.26)							
	3JH	0.227±0.06	0.22 (0.18–0.27)							
	4JH	0.223±0.06	0.22 (0.18–0.27)							
	5JH	0.226±0.06	0.21 (0.18–0.27)						0.036	17.85
	1GCT	0.231±0.06	0.22 (0.19–0.25)	0.889 (0.849–0.921)	0.007	0.020	ηp²=0.046 *p* < 0.001	1_4: *p* = 0.02, d = 0.52 1_5: *p* = 0.006, d = 0.57	0.015–0.023	7.16–9.28
	2GCT	0.225±0.04	0.23 (0.20–0.24)							
	3GCT	0.215±0.03	0.22 (0.19–0.24)							
	4GCT	0.209±0.04	0.21 (0.18–0.24)							
	5GCT	0.207±0.03	0.21 (0.18–0.23)						0.037	15.46
	1RSI	0.993±0.42	0.93 (0.70–1.24)	0.986 (0.957–0.977)	0.016	0.044	*p* = 0.127	N/A	0.117–0.133	11.35–15.28
	2RSI	1.017±0.35	0.94 (0.75–1.23)							
	3RSI	1.083±0.35	1.01 (0.83–1.29)							
	4RSI	1.088±0.33	0.99 (0.87–1.22)							
	5RSI	1.117±0.37	1.04 (0.87–1.38)						0.225	25.00
24 cm	1JH	0.245±0.06	0.24 (0.20–0.28)	0.954 (0.938–0.968)	0.005	0.014	*p* = 0.546	N/A	0.020–0.026	8.23–11.49
	2JH	0.255±0.06	0.25 (0.23–0.29)							
	3JH	0.255±0.05	0.25 (0.22–0.29)							
	4JH	0.255±0.06	0.25 (0.22–0.29)							
	5JH	0.260±0.06	0.26 (0.22–0.29)						0.041	17.37
	1GCT	0.225±0.06	0.22 (0.19–0.25)	0.899 (0.863–0.928)	0.006	0.018	ηp²=0.033 *p* = 0.005	1_4: *p* = 0.03, d = 0.49 1_5: *p* = 0.03, d = 0.50	0.014–0.024	6.77–10.11
	2GCT	0.213±0.04	0.21 (0.19–0.23)							
	3GCT	0.211±0.03	0.21 (0.19–0.23)							
	4GCT	0.205±0.03	0.20 (0.18–0.23)							
	5GCT	0.205±0.03	0.20 (0.18–0.23)						0.039	16.71
	1RSI	1.160±0.42	1.08 (0.87–1.37)	0.960 (0.946–0.972)	0.029	0.080	*p* = 0.089	N/A	0.116–0.163	9.14–15.44
	2RSI	1.226±0.34	1.14 (0.99–1.46)							
	3RSI	1.237±0.32	1.18 (1.03–1.39)							
	4RSI	1.267±0.34	1.23 (1.03–1.45)							
	5RSI	1.302±0.38	1.29 (1.03–1.52)						0.26	23.68
32 cm	1JH	0.269±0.06	0.27 (0.23–0.31)	0.959 (0.944–0.971)	0.005	0.013	*p* = 0.548	N/A	0.020–0.025	7.49–9.42
	2JH	0.277±0.06	0.28 (0.23–0.33)							
	3JH	0.282±0.06	0.28 (0.25–0.33)							
	4JH	0.281±0.07	0.28 (0.23–0.34)							
	5JH	0.284±0.07	0.29 (0.23–0.34)						0.042	15.29
	1GCT	0.224±0.06	0.21 (0.18–0.24)	0.891 (0.852–0.922)	0.006	0.018	ηp²=0.032 *p* = 0.007	1_4: *p* = 0.023, d = 0.51	0.013–0.022	5.95–9.09
	2GCT	0.213±0.04	0.21 (0.18–0.23)							
	3GCT	0.207±0.03	0.21 (0.19–0.23)							
	4GCT	0.204±0.03	0.20 (0.18–0.23)							
	5GCT	0.208±0.03	0.21 (0.19–0.23)						0.039	16.53
	1RSI	1.273±0.42	1.25 (0.96–1.49)	0.946 (0.927–0.962)	0.039	0.107	*p* = 0.089	N/A	0.140–0.170	10.33–13.78
	2RSI	1.331±0.37	1.31 (1.04–1.63)							
	3RSI	1.393±0.36	1.37 (1.11–1.64)							
	4RSI	1.412±0.41	1.37 (1.11–1.68)							
	5RSI	1.406±0.43	1.41 (1.06–1.70)						0.298	23.39

JH=jump height; GCT=ground contact time; RSI=reactive strength index; M=mean; SD=standard deviation; Median (25–75%)=median with interquartile range; ICC=intraclass correlation coefficient (95% confidence interval); SEM=standard error of measurement; MDC=minimal detectable change; ηp²=partial eta squared; d=Cohen’s d effect size; MAE=mean absolute error; MAPE=mean absolute percentage error; N/A = not applicable

Subgroup reliability coefficients were good to excellent (ICC = 0.86–0.97, 0.79–0.98, 95% CI) with only two values being below the 0.9 threshold (GCT16 (ICC = 0.86, 0.79–0.92 95%CI) and GCT32 in the inexperienced subgroup (ICC = 0.89, 0.82–0.93 95%CI)). In the direct subgroup comparison, there was an overlap of the respective inexperienced versus experienced 95% CIs. Detailed subgroup results can be reviewed in Table 2.

**Table 2:** Interday reliability metrics for drop jump performance metrics stratified for drop heights as well as previous drop jump experience accounting for descriptives, relative reliability as well as random and systematic measurement errors

		Parameter	M±SD	ICC (95% CI)	SEM	MDC	Intersubject effect	ANOVA (Main Effect)	ANOVA (Interaction)	Sig. Post-Hoc	MAE _MIN−MAX_	MAPE _MIN_MAX_
16 cm	EXP	1JH	0.263±0.062	0.948 (0.918–0.969)	0.006	0.015	*p* < 0.001, ηp²=0.367	*p* = 0.013, ηp²=0.036	*p* < 0.001, ηp²=0.076	UnExp T1_T2: *p* = 0.003, d = 0.32 T1_T3: *p* < 0.001, d = 0.46, T1_4: *p* = 0.005, d = 0.51 T1_T5: *p* = 0.006, d = 0.47	0.020–0.028	8.18–11.54
		2JH	0.258±0.053									
		3JH	0.263±0.055									
		4JH	0.252±0.062									
		5JH	0.260±0.065								0.037	14.76
	UNEXP	1JH	0.171±0.047	0.943 (0.912–0.965)	0.005	0.014					0.017–0.023	8.91–13.23
		2JH	0.187±0.046									
		3JH	0.194±0.040									
		4JH	0.197±0.040									
		5JH	0.195±0.065								0.035	20.70
	EXP	1GCT	0.216±0.047	0.926 (0.883–0.956)	0.005	0.013	*p* = 0.129	*p* < 0.001, ηp²=0.134	*p* = 0.013, ηp²=0.040	UnExp T1_T3: *p* = 0.015, d = 0.64 T1_T4: *p* = 0.001, d = 0.82 T1_T5: *p* < 0.001, d = 0.83 T2_T3: *p* = 0.01, d = 0.31 T2_T4: *p* < 0.001, d = 0.49, T2_T5: *p* < 0.001, d = 0.50	0.017–0.019	8.12–9.01
		2GCT	0.218±0.040									
		3GCT	0.212±0.037									
		4GCT	0.208±0.039									
		5GCT	0.204±0.029								0.025	11.15
	UNEXP	1GCT	0.245±0.071	0.863 (0.789–0.917)	0.009	0.025					0.014–0.028	6.85–10.15
		2GCT	0.231±0.042									
		3GCT	0.218±0.040									
		4GCT	0.211±0.033									
		5GCT	0.210±0.029								0.048	19.17
	EXP	1RSI	1.264±0.380	0.963 (0.943–0.978)	0.026	0.071	*p* < 0.001, ηp²=0.296	*p* < 0.001, ηp²=0.130	*p* < 0.001, ηp²=0.097	UnExp T1_T2: *p* = 0.008, d = 0.28 T1_T3: *p* < 0.001, d = 0.52 T1_T4: *p* < 0.001, d = 0.65 T1_T5: *p* < 0.001, d = 0.64 T2_T4: *p* = 0.002, d = 0.37 T2_T5: *p* = 0.006, d = 0.36	0.125–0.156	10.39–13.06
		2RSI	1.215±0.307									
		3RSI	1.273±0.332									
		4RSI	1.236±0.344									
		5RSI	1.301±0.378								0.20	16.45
	UNEXP	1RSI	0.751±0.299	0.956 (0.931–0.973)	0.028	0.079					0.110–0.123	11.77–18.23
		2RSI	0.841±0.284									
		3RSI	0.913±0.269									
		4RSI	0.957±0.257									
		5RSI	0.953±0.286								0.249	32.37
24 cm	EXP	1JH	0.284±0.059	0.946 (0.915–0.968)	0.006	0.015	*p* < 0.001, ηp²=0.286	*p* = 0.006, ηp²=0.041	*p* = 0.002, ηp²=0.046	UNEXP T1_T2: *p* = 0.008, d = 0.35 T1_T3: *p* = 0.019, d = 0.38 T1_T4: *p* = 0.006, d = 0.54	0.020–0.025	7.29–9.07
		2JH	0.285±0.053									
		3JH	0.284±0.050									
		4JH	0.280±0.059									
		5JH	0.285±0.064								0.040	14.78
	UNEXP	1JH	0.210±0.047	0.931 (0.894–0.958)	0.006	0.016					0.016–0.029	8.26–14.40
		2JH	0.227±0.042									
		3JH	0.229±0.040									
		4JH	0.232±0.044									
		5JH	0.237±0.043								0.042	19.56
	EXP	1GCT	0.213±0.061	0.905 (0.851–0.944)	0.006	0.016	*p* = 0.575	*p* < 0.001, ηp²=0.103	*p* = 0.013, ηp²=0.036	UNEXP T1_T2: *p* = 0.012, d = 0.48 T1_T3: *p* = 0.009, d = 0.59 T1_T4: *p* < 0.001, d = 0.76 T1_T5: *p* = 0.005, d = 0.75	0.013–0.019	5.97–8.64
		2GCT	0.210±0.035									
		3GCT	0.209±0.036									
		4GCT	0.206±0.034									
		5GCT	0.204±0.035								0.033	14.60
	UNEXP	1GCT	0.235±0.061	0.902 (0.849–0.941)	0.006	0.019					0.014–0.028	6.55–11.57
		2GCT	0.216±0.039									
		3GCT	0.212±0.030									
		4GCT	0.205±0.032									
		5GCT	0.206±0.032								0.044	18.43
	EXP	1RSI	1.388±0.402	0.960 (0.937–0.976)	0.028	0.078	*p* < 0.001, ηp²=0.205	*p* < 0.001, ηp²=0.118	*p* < 0.001, ηp²=0.076	UNEXP T1_T2: *p* < 0.001, d = 0.41 T1_T3: *p* < 0.001, d = 0.46 T1_T4: *p* < 0.001, d = 0.64 T1_T5: *p* < 0.001, d = 0.72 T2_T5: p00.028, d = 0.31	0.119–0.145	8.65–10.82
		2RSI	1.387±0.321									
		3RSI	1.390±0.317									
		4RSI	1.389±0.317									
		5RSI	1.434±0.401								0.22	16.76
	UNEXP	1RSI	0.947±0.322	0.948 (0.920–0.968)	0.033	0.093					0.109–0.177	9.43–19.39
		2RSI	1.082±0.291									
		3RSI	1.100±0.256									
		4RSI	1.157±0.293									
		5RSI	1.185±0.312								0.297	29.41
32 cm	EXP	1JH	0.295±0.061	0.942 (0.909–0.966)	0.006	0.017	*p* = 0.004, ηp²=0.093	*p* = 0.003, ηp²=0.045	*p* = 0.019, ηp²=0.033	N/A	0.022–0.027	7.90–9.46
		2JH	0.299±0.061									
		3JH	0.300±0.056									
		4JH	0.294±0.064									
		5JH	0.301±0.069								0.042	14.63
	UNEXP	1JH	0.246±0.055	0.969 (0.952–0.981)	0.004	0.011					0.016–0.024	6.23–9.77
		2JH	0.257±0.057									
		3JH	0.265±0.061									
		4JH	0.270±0.068									
		5JH	0.270±0.069								0.042	15.74
	EXP	1GCT	0.208±0.045	0.905 (0.850–0.944)	0.005	0.014	*p* = 0.094	*p* < 0.001, ηp²=0.096	*p* < 0.001, ηp²=0.053	UNEXP T1_T2: *p* = 0.041, d = 0.43 T1_T3: *p* = 0.002, d = 0.67 T1_T4: *p* < 0.001, d = 0.83 T1_T5: *p* = 0.018, d = 0.75	0.012–0.018	5.95–8.44
		2GCT	0.205±0.031									
		3GCT	0.201±0.034									
		4GCT	0.205±0.028									
		5GCT	0.207±0.028								0.031	14.27
	UNEXP	1GCT	0.237±0.067	0.886 (0.823–0.931)	0.007	0.020					0.012–0.027	5.56–10.36
		2GCT	0.221±0.040									
		3GCT	0.211±0.032									
		4GCT	0.205±0.028									
		5GCT	0.208±0.028								0.047	18.93
	EXP	1RSI	1.461±0.384	0.946 (0.914–0.968)	0.036	0.100	*p* < 0.001, ηp²=0.108	*p* < 0.001, ηp²=0.093	*p* < 0.001, ηp²=0.061	UNEXP T1_T3: *p* = 0.002, d = 0.46 T1_T4: *p* < 0.001, d = 0.63 T1_T5: *p* = 0.002, d = 0.61 T2_T4: *p* < 0.001, d = 0.39 T2_T5: *p* = 0.027, d = 0.38	0.153–0.179	10.54–13.05
		2RSI	1.484±0.355									
		3RSI	1.521±0.345									
		4RSI	1.489±0.408									
		5RSI	1.484±0.417								0.235	16.91
	UNEXP	1RSI	1.105±0.375	0.941 (0.909–0.964)	0.043	0.120					0.114–0.174	9.44–15.59
		2RSI	1.194±0.321									
		3RSI	1.279±0.343									
		4RSI	1.344±0.399									
		5RSI	1.336±0.427								0.359	29.36

JH=jump height; GCT=ground contact time; RSI=reactive strength index; EXP=experienced participants; UNEXP=inexperienced participants; M=mean; SD=standard deviation; Median (25–75%)=median with interquartile range; ICC=intraclass correlation coefficient (95% confidence interval); SEM=standard error of measurement; MDC=minimal detectable change; ηp²=partial eta squared; d=Cohen’s d effect size; MAE=mean absolute error; MAPE=mean absolute percentage error; N/A = not applicable

#### Interday systematic effects

In the overall population there were small magnitude, significant ANOVA interactions for GCT (ηp²= 0.02–0.05, *p* < 0.001–0.007) stemming from day 1 to day 4 and 5 systematic GCT shortening (*p* = 0.006–0.03, d = 0.49–0.57). No systematic effects were obtained for JH and RSI (*p* = 0.09–0.70) (see Table 1).

The experienced participant subgroup showed significantly higher baseline performance for JH and RSI (η_p_²=0.093–0.296, *p* < 0.001–0.004), but no subgroup differences for GCTs (*p* = 0.094–0.575). Small to large main effects indicated systematic performance increases over the five testing days (η_p_²=0.036–0.134, *p* < 0.001–0.013). A significant Time $\:\times\:$ Experience interaction (η_p_²=0.033–0.097, *p* < 0.001–0.019) indicated performance enhancements between day one and following testing days. In detail, in JH16, JH24, GCT24 and GCT32, there were only differences from the first test day to the second to fifth testing day (d = 0.32–0.83, *p* < 0.001–0.041). In GCT16, RSI16, RSI 24 and RSI32, also day two to day four and/or five showed significant test-retest improvements with moderate to large magnitude effect sizes (d = 0.31–0.50, *p* < 0.001–0.028). For jump height at the 32 cm drop height, no relevant post-hoc test reached the level of significance.

**Fig. 4:**
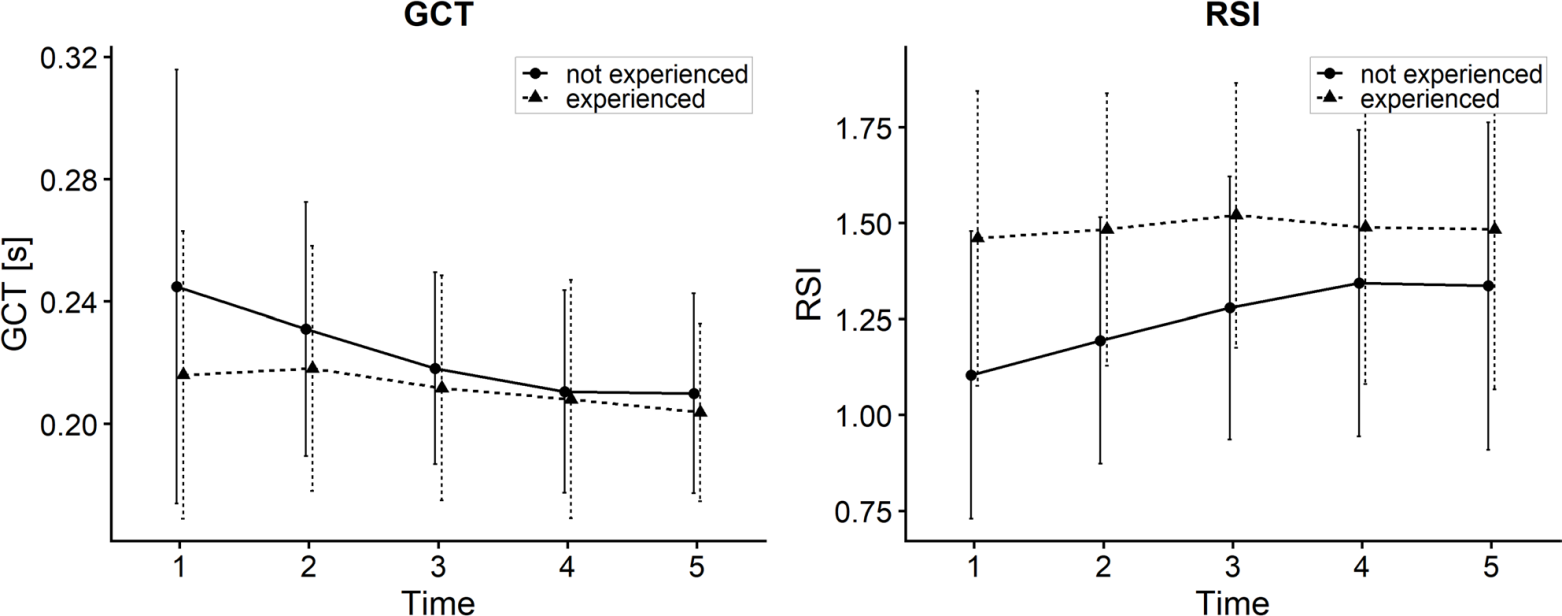
Showing mean courses with standard 95% Confidence intervals for A ground contact times (24 cm) and B the reactive strength index (32 cm) over the five testing days stratified for experience

**Fig. 5:**
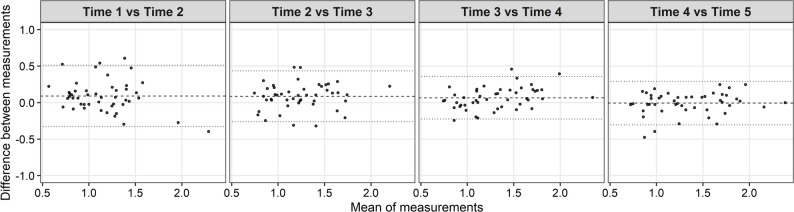
Provides agreement analyses for the RSI (32 cm) for the four testing day comparisons for the inexperienced subgroup

More detailed results are presented in Table 2. Test-retest mean courses are exemplary illustrated in Fig. 4 for the 32 cm drop height RSI and GCTs in the 24 cm drop height (Fig. 5).

#### Random measurement error analysis

The MAE and MAPE day-to-day comparisons for JH and GCTs were consistently around 10% with the lowest MAPE reported for GCTs (5.95%) and the highest for jump height (24 cm drop height) (11.49%). In contrast, the RSIs were > 10% with a maximum of 15.44% in 24 cm drop height testing. The SEM for jump height ranged between 0.003 and 0.005 cm, GCT SEMs were between 0.006 and 0.007s and the RSI showed SEMs between 0.016 and 0.039 which increased with enhancing the drop height.

For individual parameters, subgroup differences were obtained. In the inexperienced group the GCT16 SEM was with 0.009s close to double as high as the 0.005s in the experienced group. Nevertheless, for most other parameters, the differences were smaller to non-existing (e.g. JH16, JH24, GCT24, GCT32, RSI 16). Larger subgroup specific differences were partly obtained when considering the MAPE. For example, the RSI32 day one to day five comparison showed a MAPE of 17% in the experienced but about 30% in the inexperienced group. Detailed random error quantifications can be reviewed in Table 2).

#### Systematic errors and minimal detectable change

For GCT 16 and 24, significant systematic errors encompassed the MDC. No such effect was found for GCT32. Although for some other parameters (e.g. JH24 and 32) the MDC was below the systematic day 1 to day 5 performance improvement, the ANOVA indicated that this increase was not statistically significant (see Table 1 for more detailed results).

While no systematic bias reached the level of significance in the experienced subgroup, in the inexperienced group, the systematic bias (where significant) exceeded the MDC (by far). For instance, in JH16, the jumping height increased from 0.171 cm in test day 1 to 0.195 cm in test day 5; the MDC was stated with 0.014 cm (see Table 2 for more detailed results).

## Discussion

This study investigated the influence of previous DJ experience on habituation effects and intra- and interday stability of RSI, jump height, and GCT across five testing days at three drop heights (16, 24, and 32 cm). In line with earlier research [40, 41], jump height and RSI showed good-to-excellent relative reliability (ICC > 0.90), whereas GCT was less reliable (lowest ICC = 0.54). Within each testing session, intraday reliability metrics indicated systematic test-to-test improvements for only some parameters, and measurement errors were mainly attributable to random variation.

In contrast, interday comparisons were affected by both systematic and random errors, particularly in inexperienced participants, while experienced participants demonstrated higher performance and greater stability, characterized by the absence of systematic bias and lower long-term MAPEs. In contrast, the inexperienced counterparts exhibited larger MAPEs and significant day-to-day performance increases which were of clinical/practical relevance, as systematic effects surpassed the MDC. Among other explanations, these effects could be attributed to task habituation effects in the inexperienced subgroup, while the absence of significant test-retest mean differences in the experienced subgroup could potentially be attributed to already familiarized participants.

### Systematic errors and internal data validity

Physical activity diagnostics are prone to task habituation [10, 42]. Habituation or practice effects are reflected in test-retest performance improvements that can be attributed to task familiarization with a previously unknown testing condition as in test-retest designs with no intervention applied between tests. These effects were shown in strength testing. The present results regarding systematic effects are in accordance with previous literature.

Ploutz-Snyder & Giamis [20] reported that young, untrained participants required 3–4 sessions (12 ± 5% increase) and older women up to nine sessions (22 ± 4%) to eliminate significant systematic effects. Similarly, 2–3 familiarization sessions were recommended to reduce habituation in other strength tests [21, 43]. Ritti-Dias et al. [19] observed an 11.2% habituation effect in the inexperienced subgroup, while similar to the present study, the experienced participants showed no meaningful task habituation in 1 repetition maximum (RM) testing. However, evaluating experience is problematic. Drake et al. [44] showed that even with participants with approximately 4 years of resistance training experience, three familiarization sessions were still required to achieve stable testing conditions in isometric squat tests. These results were confirmed by Kite & Callaway [21] who showed that over three testing days with three trials per day, ICCs improved over time and testing results stabilized in the isometric squat test.

Interestingly, task habituation studies in DJ research are scarce across the literature. Instead, it is common practice to refer to the DJ as a reliable and valid test (e.g. [45, 46]. Considering a test to be reliable, per se, must be questioned from a scientific perspective [47]. Adopting reliability metrics from previous research was described as “reliability induction” and was questioned in terms of scientific rigor as the magnitude of measurement errors and reliability critically depends on the participants and standardization, which cannot be transferred between studies [48]. Accordingly, numerous factors might influence habituation effects, with previous task exposure and the understanding of test execution might meaningfully influence the magnitude of task-familiarization effects on performance outcomes. These are commonly quantified via t-tests or ANOVA [10]. To our knowledge, this is the first study to provide a detailed measurement-error analysis of the influence of previous task exposure.

### Random errors and internal data validity

Previous research on habituation effects has focused on systematic error, whereas the influence on random error components remains underexplored. In general, MAPEs ranged between 30% MAPE for the RSI (see Table 2). These results are partly in accordance with previous studies showing that the “absolute reliability” of the RSI showed meaningful measurement errors, with a variability coefficient of ≥ 12% despite excellent ICCs ≥ 0.92 [49]. Large random errors might be attributable to biological variability as one main contributor to heterogeneity in movement strategies. Other potential sources, such as laboratory temperature, time of day, and fatigue induced by previous activity, were controlled via exclusion criteria.

The comparatively large random measurement errors within this work must be considered a key finding. Even though a reduction of the random error could be reached in some parameters after extensive habituation within this study, these familiarization sessions were still insufficient to reduce the random error below an often-applied critical threshold of 10% [50–52]. Especially when including inexperienced or untrained participants, the present results underline the relevance of accounting for random error quantification in future studies. Although athletes accustomed to the DJ may be able to achieve GCTs < 250ms, numerous studies have been performed with untrained participants without quantifying the random error. Although the 30% day-1-to-day-5 error margin found in this work might be population-specific and may differ in other studies, future research is encouraged to quantify random measurement errors in addition to widely implemented reliability coefficients, as relevant performance variability cannot be ruled out and the sole reporting of ICCs can be misleading [10, 29, 53].

Beyond the relevance of a (up to) 30% MAPE, per se, the results of this study indicate potential influence of task habituation on measurement precision, which was reflected in a reduction of the random error. The random measurement error development over several testing times deserves specific attention as previous studies focused on systematic errors when evaluating habituation effects. Therefore, this study is the first showing that not just the systematic, but also the random error can be affected by task habituation in DJ (see Fig. 5), especially in the inexperienced subgroup.

However, the MAPE quantification approach has limitations in the presence of systematic errors and can overestimate the random error, as it is based on absolute test-retest differences. Accordingly, the 30% MAPE observed in inexperienced participants should be interpreted with caution. Nevertheless, compared with the experienced group, the measurement error was still meaningfully higher.

### The reliability – validity interaction: Potential implications for future research

The relevance of reliability for the meaningful interpretation of research results was outlined in numerous articles [9, 10, 29, 32, 53]. As shown by the present results, the ability to adhere with cornerstones of DJ testing can be influenced by performance level at test entry and previous task exposure. Many inexperienced participants were unable to meet the GCT criterion on test day 1 but achieved valid performance after repeated testing on day 5. This is critical because short GCTs are a pre-requisite for effective SSC utilization. To potentiate performance via DJ, literature has outlined that attached cross-bridges during the eccentric phase might store energy in the muscle to avoid excessive muscle lengthening and maintain a relatively constant muscle length, while lengthening and shortening might occur primarily in tendinous tissue, such as the Achilles tendon [54]. Given the importance to adhere to these guidelines reliably, it could be questioned whether the test was valid to evaluate the intended physiological. Accordingly, reliability could have impact on validity of the DJ testing.

Numerous previous articles did not report GCTs at all [49, 55] complicating judgments about the validity of the results and reducing transparency in result reporting. Other studies referred to DJ performance even though GCTs meaningfully surpassed the 250ms threshold [56, 57]. This issue might, in part, be attributable to insufficient task habituation. Since this study indicated that GCTs decreased over five familiarization days and most participants were afterwards able to realize shorter GCTs, familiarization might not just improve reliability and precision of the test but could also help participants to adhere to the oftentimes proposed 250ms threshold.

Based on the presented data, conclusions that the DJ is a reliable test, per se [57, 58], seems questionable, as performance as well as task habituation might depend on several factors; previous task experience might be one moderator. Therefore, authors should report relevant key performance characteristics such as GCTs and show that the recruited sample was able to perform DJ appropriately. Here, a detailed measurement error analysis that supplements the commonly performed ICC quantification can help to further improve transparency of results reporting.

## Limitations

Several limitations should be considered when interpreting the study results. Although the sample size of 89 participants was comparatively large, subgroup splitting reduced the sample into two slightly unequal groups, which widened the 95% CIs. The different participant numbers per group also resulted from dropouts. Although both subgroups included > 40 participants, further separation to evaluate additional influencing factors, such as sex, would further reduce subgroup sample sizes and result in underpowered outcomes. Especially in females, menstrual-cycle-related effects could have affected the results, however, data on hormonal status or menstrual cycle phase were not collected. Although some potential effects of circadian rhythm deviations, fatigue, or daily form were controlled by scheduling participants within a maximum deviation of ± 1 h each day, physiological marker analyses before testing could improve the control of potential influences in future studies. In addition, hydration and nutritional status, could provide additional value.

The intraday reliability metrics, consisting of three data points per participant, drop height, and test day, resulted in a series of ANOVAs [59]. In addition, the sample consisted of physical education and sport science students, which limits external validity to other populations, such as high-performance athletes.

Subgroup splitting was based on previous experience with DJ tasks and the ability to adhere to the 250ms GCT threshold, with criteria synthesized from the literature. The criteria are vague, as no accepted guidelines on how to discriminate between experienced and inexperienced participants exist, which risks definitional overlap and highlights the need for guidelines to classify experience in future drop jump research.

Not all variables were normally distributed; however, ANOVA is considered robust for *n* > 30, a criterion that was met, and results were confirmed using non-parametric tests. Moreover, absolute error metrics are independent of distributional assumptions and p-values, and ICC precision was evaluated using 95% CIs.

In this study, in line with previous research, the mean of the repeated trials per day was used for the analysis of interday reliability [18], as aggregation of multiple attempts is known to attenuate random error variance and to provide a stable estimate of habitual performance. While some studies have used maximal values to calculate interday reliability [17], maximal performance represents a different construct and appears, due to the mathematical properties of maximal values, to be more sensitive to learning- and practice-related fluctuations, which may be reflected in lower reliability indices.

## Practical applications

In accordance with previous study results, task experience had a significant influence on task habituation and learning curves in DJ. Researchers are encouraged to extend common reliability-coefficient-based reporting using ICCs with additional metrics that account for systematic and random errors, in order to transparently report whether habituation effects could potentially bias interpretability. Sample-specific reliability and measurement errors were found in this study, with a day-1-to-day-5 MAPE of up to 30%, despite reliability coefficients being mostly good to excellent.

These results indicate that sufficient DJ task habituation can require extensive familiarization and appropriate task learning, while measurement errors remain inherent. This specifically applies to participants who are unfamiliar with DJ tasks or generally untrained, as systematic and random measurement errors were found to be pronounced in this population. Accordingly, researchers and practitioners are encouraged to carefully control habituation effects to transparently address potential sources of bias when interpreting diagnostic results.

## Supplementary Information

Supplementary Material 1.

## Data Availability

The data that support the findings of this study are available from the corresponding author upon reasonable request.
