# Supplementary material for: Does task experience moderate habituation effects in drop jumps? - An intra- and interday reliability and measurement error analysis
Source: BMC Sports Sci Med Rehabil. 2026 Jun 29;18:300. doi: 10.1186/s13102-026-01833-3 (PMC13321856; doi:10.1186/s13102-026-01833-3)
Supplement: Supplementary file 1 — Supplementary Material 1. [file 13102_2026_1833_MOESM1_ESM.docx]

**SUPPLEMENTAL MATERIAL**

**Table S1** descriptives for within day reactive strength values with intrasession reliability metrics including relative reliability, the systematic and random error

| Day | Parameter | (M±SD) | Median (25% - 75%) | ICC (95% CI) | SEM | MDC | ANOVA | MAE_Min-Max_ | MAPE_Min-Max_ (in%) |
| --- | --- | --- | --- | --- | --- | --- | --- | --- | --- |
| Day 1 | JH16 _T1 | 0.210±0.07 | 0.21 (0.15 – 0.27) | 0.966 (0.951 – 0.976) | 0.003 | 0.010 | p=0.623 | 0.023 – 0.025 | 11.72 – 13.13 |
|  | JH16 _T2 | 0.215±0.07 | 0.21 (0.16 – 0.27) |  |  |  |  |  |  |
|  | JH16 _T3 | 0.219±0.07 | 0.22 (0.16 – 0.26) |  |  |  |  |  |  |
|  | GCT16 _T1 | 0.232±0.05 | 0.23 (0.20 – 0.25) | 0.813 (0.734 – 0.871) | 0.010 | 0.027 | p=0.889 | 0.024 – 0.032 | 9.73 – 11.92 |
|  | GCT16 _T2 | 0.228±0.06 | 0.22 (0.19 – 0.24) |  |  |  |  |  |  |
|  | GCT16 _T3 | 0.233±0.10 | 0.21 (0.19 – 0.25) |  |  |  |  |  |  |
|  | RSI16 _T1 | 0.954±0.43 | 0.88 (0.61 – 1.24) | 0.965 (0.951 – 0.976) | 0.022 | 0.060 | p=0.454 | 0.118 – 0.164 | 13.78 – 17.69 |
|  | RSI16 _T2 | 1.008±0.44 | 0.92 (0.70 – 1.27) |  |  |  |  |  |  |
|  | RSI16 _T3 | 1.035±0.45 | 1.02 (0.68 – 1.33) |  |  |  |  |  |  |
|  | JH24 _T1 | 0.243±0.07 | 0.24 (0.20 – 0.28) | 0.970 (0.957 – 0.979) | 0.003 | 0.007 | p=0.959 | 0.019 – 0.020 | 8.79 – 10.29 |
|  | JH24 _T2 | 0.246±0.07 | 0.24 (0.21 – 0.27) |  |  |  |  |  |  |
|  | JH24 _T3 | 0.246±0.07 | 0.25 (0.21 – 0.28) |  |  |  |  |  |  |
|  | GCT24 _T1 | 0.224±0.06 | 0.22 (0.18 – 0.25) | 0.945 (0.922 – 0.962) | 0.004 | 0.010 | p=0.966 | 0.018 – 0.020 | 7.46 – 9.66 |
|  | GCT24 _T2 | 0.226±0.05 | 0.22 (0.19 – 0.25) |  |  |  |  |  |  |
|  | GCT24 _T3 | 0.225±0.06 | 0.22 (0.19 – 0.24) |  |  |  |  |  |  |
|  | RSI24 _T1 | 1.167±0.43 | 1.08 (0.85 – 1.46) | 0.976 (0.965 – 0.983) | 0.013 | 0.038 | p=0.970 | 0.105 – 0.117 | 9.83 – 10.92 |
|  | RSI24 _T2 | 1.152±0.42 | 1.10 (0.88 – 1.38) |  |  |  |  |  |  |
|  | RSI24 _T3 | 1.164±0.45 | 1.12 (0.87 – 1.38) |  |  |  |  |  |  |
|  | JH32 _T1 | 0.266±0.06 | 0.27 (0.22 – 0.31) | 0.973 (0.962 – 0.981) | 0.002 | 0.007 | p=0.966 | 0.018 – 0.019 | 6.74 – 7.92 |
|  | JH32 _T2 | 0.270±0.06 | 0.27 (0.23 – 0.31) |  |  |  |  |  |  |
|  | JH32 _T3 | 0.272±0.07 | 0.272 (0.23 – 0.32) |  |  |  |  |  |  |
|  | GCT32 _T1 | 0.224±0.06 | 0.22 (0.19 – 0.25) | 0.972 (0.960 – 0.981) | 0.002 | 0.006 | p=0.947 | 0.014 – 0.016 | 5.924 – 6.82 |
|  | GCT32 _T2 | 0.222±0.60 | 0.21 (0.18 – 0.24) |  |  |  |  |  |  |
|  | GCT32 _T3 | 0.225±0.06 | 0.21 (0.19 – 0.25) |  |  |  |  |  |  |
|  | RSI32_T1 | 1.262±0.41 | 1.21 (0.96 – 1.48) | 0.978 (0.969 – 0.985) | 0.013 | 0.035 | p=0.914 | 0.100 – 0.113 | 8.50 – 9.43 |
|  | RSI32 _T2 | 1.289±0.43 | 1.27 (0.98 – 1.54) |  |  |  |  |  |  |
|  | RSI32 _T3 | 1.276±0.43 | 1.27 (0.97 – 1.53) |  |  |  |  |  |  |
| Day 2 | JH16 _T1 | 0.217±0.06 | 0.22 (0.17 – 0.26) | 0.920 (0.889 – 0.944) | 0.004 | 0.012 | p=0.772 | 0.016 – 0.022 | 7.67 – 10.47 |
|  | JH16 _T2 | 0.222±0.06 | 2.1 (0.18 – 0.27) |  |  |  |  |  |  |
|  | JH16 _T3 | 0.223±0.06 | 0.22 (0.18 – 0.26) |  |  |  |  |  |  |
|  | GCT16 _T1 | 0.230±0.05 | 0.23 (0.20 – 0.25) | 0.784 (0.710 – 0.844) | 0.007 | 0.019 | p=0.414 | 0.015 – 0.023 | 6.33 – 9.75 |
|  | GCT16 _T2 | 0.224±0.04 | 0.22 (0.19 – 0.25) |  |  |  |  |  |  |
|  | GCT16 _T3 | 0.221±0.04 | 0.22 (0.20 – 0.25) |  |  |  |  |  |  |
|  | RSI16 _T1 | 0.988±0.36 | 0.94 (0.73 – 1.22) | 0.896 (0.857 – 0.927) | 0.031 | 0.085 | p=0.552 | 0.098 – 0.135 | 10.28 – 14.24 |
|  | RSI16 _T2 | 1.034±0.37 | 0.97 (0.75 – 1.25) |  |  |  |  |  |  |
|  | RSI16 _T3 | 1.043±0.35 | 1.03 (0.76 – 1.27) |  |  |  |  |  |  |
|  | JH24 _T1 | 0.255±0.06 | 0.25 (0.22 – 0.29) | 0.918 (0.886 – 0.942) | 0.004 | 0.010 | p=0.942 | 0.016 – 0.017 | 6.45 – 8.35 |
|  | JH24 _T2 | 0.253±0.05 | 0.25 (0.22 – 0.29) |  |  |  |  |  |  |
|  | JH24 _T3 | 0.256±0.06 | 0.25 (0.23 – 0.29) |  |  |  |  |  |  |
|  | GCT24 _T1 | 0.212±0.04 | 0.21 (0.18 – 0.23) | 0.820 (0.757 – 0.872) | 0.006 | 0.015 | p=0.912 | 0.016 – 0.017 | 7.23 – 9.14 |
|  | GCT24 _T2 | 0.214±0.04 | 0.22 (0.19 – 0.23) |  |  |  |  |  |  |
|  | GCT24 _T3 | 0.214±0.04 | 0.22 (0.19 – 0.24) |  |  |  |  |  |  |
|  | RSI24 _T1 | 1.235±0.34 | 1.17 (1.00 – 1.47) | 0.935 (0.910 – 0.955) | 0.020 | 0.055 | p=0.920 | 0.093 – 0.109 | 8.09 – 9.56 |
|  | RSI24 _T2 | 1.216±0.34 | 1.16 (1,00 – 1.40) |  |  |  |  |  |  |
|  | RSI24 _T3 | 1.232±0.36 | 1.18 (1.01 – 1.45) |  |  |  |  |  |  |
|  | JH32 _T1 | 0.274±0.06 | 0.27 (0.23 – 0.33) | 0.933 (0.907 – 0.954) | 0.004 | 0.010 | p=0.871 | 0.017 – 0.018 | 6.20 – 6.77 |
|  | JH32 _T2 | 0.279±0.07 | 0.29 (0.24 – 0.32) |  |  |  |  |  |  |
|  | JH32 _T3 | 0.277±0.06 | 0.27 (0.24 – 0.33) |  |  |  |  |  |  |
|  | GCT32 _T1 | 0.212±0.04 | 0.21 (0.18 – 0.24) | 0.872 (0.824 – 0.910) | 0.004 | 0.011 | p=0.912 | 0.013 – 0.016 | 6.21 – 7.26 |
|  | GCT32 _T2 | 0.215±0.04 | 0.21 (0.19 – 0.23) |  |  |  |  |  |  |
|  | GCT32 _T3 | 0.213±0.04 | 0.22 (0.18 – 0.23) |  |  |  |  |  |  |
|  | RSI32_T1 | 1.326±0.36 | 1.27 (1.04 – 1.60) | 0.918 (0.886 – 0.942) | 0.027 | 0.075 | p=0.970 | 0.111 – 0.132 | 8.68 – 10.41 |
|  | RSI32 _T2 | 1.337±0.38 | 1.32 (1.06 – 1.63) |  |  |  |  |  |  |
|  | RSI32 _T3 | 1.338±0.39 | 1.30 (0.99 – 1.62) |  |  |  |  |  |  |
| Day 3 | JH16 _T1 | 0.224±0.06 | 0.22 (0.18 – 0.27) | 0.895 (0.855 – 0.926) | 0.005 | 0.014 | p=0.764 | 0.017 – 0.022 | 7.68 – 9.83 |
|  | JH16 _T2 | 0.226±0.06 | 0.22 (0.18 – 0.27) |  |  |  |  |  |  |
|  | JH16 _T3 | 0.230±0.06 | 0.22 (0.17 – 0.28) |  |  |  |  |  |  |
|  | GCT16 _T1 | 0.219±0.04 | 0.22 (0.19 – 0.24) | 0.787 (0.714 – 0.847) | 0.006 | 0.018 | p=0.294 | 0.016 – 0.020 | 7.18 – 7.79 |
|  | GCT16 _T2 | 0.216±0.04 | 0.22 (0.19 – 0.24) |  |  |  |  |  |  |
|  | GCT16 _T3 | 0.210±0.03 | 0.21 (0.19 – 0.23) |  |  |  |  |  |  |
|  | RSI16 _T1 | 1.058±0.35 | 0.99 (0.78 – 1.28) | 0.899 (0.860 – 0.929) | 0.031 | 0.085 | p=0.533 | 0.107 – 0.134 | 10.51 – 12.72 |
|  | RSI16 _T2 | 1.081±0.37 | 0.98 (0.80 – 1.29) |  |  |  |  |  |  |
|  | RSI16 _T3 | 1.118±0.36 | 1.03 (0.82 – 1.32) |  |  |  |  |  |  |
|  | JH24 _T1 | 0.252±0.06 | 0.25 (0.22 – 0.28) | 0.921 (0.890 – 0.945) | 0.004 | 0.010 | p=0.755 | 0.015 – 0.017 | 6.37 – 7.07 |
|  | JH24 _T2 | 0.255±0.05 | 0.25 (0.22 – 0.30) |  |  |  |  |  |  |
|  | JH24 _T3 | 0.258±0.05 | 0.26 (0.23 – 0.29) |  |  |  |  |  |  |
|  | GCT24 _T1 | 0.211±0.04 | 0.21 (0.19 – 0.23) | 0.846 (0.790 – 0.891) | 0.004 | 0.012 | p=0.974 | 0.014 – 0.015 | 6.63 – 7.22 |
|  | GCT24 _T2 | 0.210±0.03 | 0.21 (0.19 – 0.24) |  |  |  |  |  |  |
|  | GCT24 _T3 | 0.210±0.04 | 0.21 (0.18 – 0.23) |  |  |  |  |  |  |
|  | RSI24 _T1 | 1.224±0.33 | 1.17 (1.02 – 1.41) | 0.907 (0.871 – 0.935) | 0.026 | 0.072 | p=0.768 | 0.109 | 8.99 – 9.29 |
|  | RSI24 _T2 | 1.236±0.33 | 1.17 (1.02 – 1.39) |  |  |  |  |  |  |
|  | RSI24 _T3 | 1.259±0.33 | 1.19 (1.03 – 1.42) |  |  |  |  |  |  |
|  | JH32 _T1 | 0.280±0.06 | 0.28 (0.25 – 0.33) | 0.931 (0.904 – 0.952) | 0.003 | 0.010 | p=0.934 | 0.015 – 0.018 | 5.88 – 6.75 |
|  | JH32 _T2 | 0.282±0.06 | 0.28 (0.25 – 0.33) |  |  |  |  |  |  |
|  | JH32 _T3 | 0.283±0.06 | 0.29 (0.24 – 0.34) |  |  |  |  |  |  |
|  | GCT32 _T1 | 0.206±0.03 | 0.21 (0.18 – 0.23) | 0.858 (0.806 – 0.899) | 0.004 | 0.012 | p=0.991 | 0.013 – 0.015 | 6.20 – 7.33 |
|  | GCT32 _T2 | 0.207±0.04 | 0.20 (0.19 – 0.23) |  |  |  |  |  |  |
|  | GCT32 _T3 | 0.206±0.03 | 0.20 (0.18 – 0.23) |  |  |  |  |  |  |
|  | RSI32_T1 | 1.384±0.36 | 1.38 (1.10 – 1.60) | 0.919 (0.887 – 0.943) | 0.026 | 0.073 | p=0.926 | 0.105 – 0.130 | 8.29 – 9.31 |
|  | RSI32 _T2 | 1.397±0.38 | 1.39 (1.13 – 1.68) |  |  |  |  |  |  |
|  | RSI32 _T3 | 1.406±0.39 | 1.39 (1.13 – 1.71) |  |  |  |  |  |  |
| Day 4 | JH16 _T1 | 0.222±0.06 | 0.21 (0.19 – 0.27) | 0.806 (0.738 – 0.861) | 0.008 | 0.021 | p=0.953 | 0.018 – 0.023 | 9.50 – 14.16 |
|  | JH16 _T2 | 0.224±0.06 | 0.22 (0.18 – 0.26) |  |  |  |  |  |  |
|  | JH16 _T3 | 0.221±0.07 | 0.22 (0.18 – 0.26) |  |  |  |  |  |  |
|  | GCT16 _T1 | 0.212±0.04 | 0.21 (0.19 – 0.24) | 0.617 (0.509 – 0.714) | 0.009 | 0.026 | p=0.674 | 0.016 – 0.022 | 8.13 – 12.82 |
|  | GCT16 _T2 | 0.209±0.04 | 0.21 (0.18 – 0.24) |  |  |  |  |  |  |
|  | GCT16 _T3 | 0.207±0.05 | 0.21 (0.18 – 0.24) |  |  |  |  |  |  |
|  | RSI16 _T1 | 1.080±0.35 | 1.01 (0.82 – 1.21) | 0.590 (0.477 – 0.691) | 0.077 | 0.214 | p=0.704 | 0.106 – 0.178 | 11.32 – 16.14 |
|  | RSI16 _T2 | 1.092±0.35 | 1.04 (0.86 – 0.12) |  |  |  |  |  |  |
|  | RSI16 _T3 | 1.131±0.55 | 1.02 (0.86 – 1.29) |  |  |  |  |  |  |
|  | JH24 _T1 | 0.249±0.07 | 0.25 (0.21 – 0.29) | 0.799 (0.729 – 0.855) | 0.007 | 0.021 | p=0.524 | 0.016 – 0.024 | 6.60 – 13.11 |
|  | JH24 _T2 | 0.258±0.05 | 0.26 (0.23 – 0.29) |  |  |  |  |  |  |
|  | JH24 _T3 | 0.257±0.06 | 0.26 (0.22 – 0.29) |  |  |  |  |  |  |
|  | GCT24 _T1 | 0.204±0.05 | 0.21 (0.19 – 0.24) | 0.539 (0.420 – 0.650) | 0.010 | 0.027 | p=0.933 | 0.014 – 0.021 | 6.53 – 12.64 |
|  | GCT24 _T2 | 0.206±0.03 | 0.20 (0.18 – 0.23) |  |  |  |  |  |  |
|  | GCT24 _T3 | 0.206±0.03 | 0.21 (0.19 – 0.23) |  |  |  |  |  |  |
|  | RSI24 _T1 | 1.232±0.36 | 1.20 (1.00 – 1.41) | 0.882 (0.838 – 0.917) | 0.034 | 0.094 | p=0.519 | 0.116- 0.132 | 9.48 – 12.28 |
|  | RSI24 _T2 | 1.290±0.36 | 1.23 (1.02 – 1.52) |  |  |  |  |  |  |
|  | RSI24 _T3 | 1.279±0.37 | 1.25 (1.05 – 1.47) |  |  |  |  |  |  |
|  | JH32 _T1 | 0.280±0.07 | 0.27 (0.23 – 0.33) | 0.960 (0.944 – 0.972) | 0.002 | 0.007 | p=0.943 | 0.014 – 0.016 | 5.32 – 6.26 |
|  | JH32 _T2 | 0.281±0.07 | 0.28 (0.22 – 0.33) |  |  |  |  |  |  |
|  | JH32 _T3 | 0.283±0.07 | 0.28 (0.23 – 0.34) |  |  |  |  |  |  |
|  | GCT32 _T1 | 0.204±0.03 | 0.21 (0.18 – 0.23) | 0.852 (0.798 – 0.895) | 0.004 | 0.011 | p=0.923 | 0.012 – 0.014 | 5.92 – 6.55 |
|  | GCT32 _T2 | 0.205±0.03 | 0.20 (0.18 – 0.23) |  |  |  |  |  |  |
|  | GCT32 _T3 | 0.203±0.03 | 0.20 (0.18 – 0.23) |  |  |  |  |  |  |
|  | RSI32_T1 | 1.410±0.41 | 1.35 (1.09 – 1.71) | 0.950 (0.930 – 0.965) | 0.018 | 0.051 | p=0.885 | 0.097 – 0.112 | 7.26 – 8.49 |
|  | RSI32 _T2 | 1.402±0.41 | 1.39 (1.08 – 1.67) |  |  |  |  |  |  |
|  | RSI32 _T3 | 1.432±0.43 | 1.42 (1.09 – 1.75) |  |  |  |  |  |  |
| Day 5 | JH16 _T1 | 0.221±0.06 | 0.21 (0.17 – 0.28) | 0.949 (0.929 – 0.965) | 0.003 | 0.008 | p=0.638 | 0.013 – 0.017 | 6.52 – 8.12 |
|  | JH16 _T2 | 0.226±0.07 | 0.20 (0.18 – 0.27) |  |  |  |  |  |  |
|  | JH16 _T3 | 0.230±0.06 | 0.22 (0.19 – 0.27) |  |  |  |  |  |  |
|  | GCT16 _T1 | 0.209±0.03 | 0.21 (0.18 – 0.23) | 0.797 (0.727 – 0.854) | 0.005 | 0.015 | p=0.541 | 0.012 – 0.017 | 5.80 – 8.04 |
|  | GCT16 _T2 | 0.208±0.03 | 0.21 (0.18 – 0.23) |  |  |  |  |  |  |
|  | GCT16 _T3 | 0.204±0.03 | 0.21 (0.18 – 0.22) |  |  |  |  |  |  |
|  | RSI16 _T1 | 1.090±0.38 | 1.01 (0.80 – 1.33) | 0.924 (0.894 – 0.947) | 0.026 | 0.071 | p=0.458 | 0.109 – 0.126 | 10.31 – 11.99 |
|  | RSI16 _T2 | 1.113±0.38 | 1.03 (0.86 – 1.33) |  |  |  |  |  |  |
|  | RSI16 _T3 | 1.160±0.39 | 1.08 (0.89 – 1.40) |  |  |  |  |  |  |
|  | JH24 _T1 | 0.257±0.06 | 0.26 (0.22 – 0.29) | 0.936 (0.911 – 0.956) | 0.003 | 0.009 | p=0.865 | 0.014 – 0.017 | 5.78 – 6.71 |
|  | JH24 _T2 | 0.260±0.06 | 0.26 (0.21 – 0.30) |  |  |  |  |  |  |
|  | JH24 _T3 | 0.262±0.06 | 0.26 (0.22 – 0.30) |  |  |  |  |  |  |
|  | GCT24 _T1 | 0.207±0.03 | 0.20 (0.18 – 0.23) | 0.837 (0.779 – 0.884) | 0.004 | 0.012 | p=0.683 | 0.013 – 0.015 | 6.25 – 7.09 |
|  | GCT24 _T2 | 0.206±0.04 | 0.21 (0.18 – 0.23) |  |  |  |  |  |  |
|  | GCT24 _T3 | 0.202±0.04 | 0.20 (0.18 – 0.22) |  |  |  |  |  |  |
|  | RSI24 _T1 | 1.281±0.38 | 1.27 (1.02 – 1.51) | 0.936 (0.911 – 0.955) | 0.021 | 0.059 | p=0.662 | 0.099 – 0.118 | 7.95 – 9.96 |
|  | RSI24 _T2 | 1.302±0.39 | 1.29 (1.03 – 1.54) |  |  |  |  |  |  |
|  | RSI24 _T3 | 1.333±0.39 | 1.34 (1.04 – 1.58) |  |  |  |  |  |  |
|  | JH32 _T1 | 0.284±0.07 | 0.28 (0.23 – 0.33) | 0.943 (0.921 – 0.961) | 0.003 | 0.009 | p=0.912 | 0.014 – 0.019 | 5.23 – 7.69 |
|  | JH32 _T2 | 0.283±0.07 | 0.28 (0.22 – 0.34) |  |  |  |  |  |  |
|  | JH32 _T3 | 0.287±0.07 | 0.28 (0.23 – 0.34) |  |  |  |  |  |  |
|  | GCT32 _T1 | 0.207±0.03 | 0.20 (0.19 – 0.23) | 0.813 (0.747 – 0.866) | 0.005 | 0.014 | p=0.665 | 0.014 – 0.016 | 6.62 – 7.55 |
|  | GCT32 _T2 | 0.206±0.03 | 0.20 (0.19 – 0.23) |  |  |  |  |  |  |
|  | GCT32 _T3 | 0.210±0.03 | 0.21 (0.18 – 0.23) |  |  |  |  |  |  |
|  | RSI32_T1 | 1.405±0.45 | 1.35 (1.07 – 1.73) | 0.917 (0.885 – 0.942) | 0.029 | 0.082 | p=0.994 | 0.127 – 0.131 | 9.74 – 10.43 |
|  | RSI32 _T2 | 1.411±0.43 | 1.44 (1.08 – 1.69) |  |  |  |  |  |  |
|  | RSI32 _T3 | 1.412±0.44 | 1.39 (1.05 – 1.75) |  |  |  |  |  |  |

*Legend: JH = jump height; GCT = ground contact time; RSI = reactive strength index; M = mean; SD = standard deviation;ICC = intraclass correlation coefficient; SEM = standard error of measurement; MDC = minimal detectable change; MAE = mean absolute error; MAPE = mean absolute percentage error; T1-T5 = testing days 1 to 5*

**Table S2** descriptives for within day reactive strength values with intrasession reliability metrics including relative reliability, the systematic and random error stratified for experience

| Day | EXP | Parameter | (M±SD) | Median (25% - 75%) | ICC (95% CI) | ANOVA (Interaction | Post-Hoc | MAE_Min-Max_ | MAPE_Min-Max_ (in%) |
| --- | --- | --- | --- | --- | --- | --- | --- | --- | --- |
| Day 1 | EXP* | JH16 _T1 | 0.264±0.058 | 0.274 (0.218 – 0.294 | 0.971 (0.952 – 0.984) | ηp²=0.049  p=0.012 | UNEXP  T1_2: p=0.029, d=0.26  T1_T3: p=0.003, d=0.31 | 0.017 – 0.023 | 13.70 – 18.18 |
|  |  | JH16 _T2 | 0.260±0.065 | 0.264 (0.224 – 0.293) |  |  |  |  |  |
|  |  | JH16 _T3 | 0.266±0.066 | 0.265 (0.220 – 0.290) |  |  |  |  |  |
|  | UNEXP* | JH16 _T1 | 0.160±0.048 | 0.152 (0.120 – 0.190) | 0.902 (0.840 – 0.942) |  |  | 0.023 – 0.03 | 7.47 – 9.51 |
|  |  | JH16 _T2 | 0.175±0.055 | 0.169 (0.133 – 0.203) |  |  |  |  |  |
|  |  | JH16 _T3 | 0.178±0.066 | 0.172 (0.137 – 0.200) |  |  |  |  |  |
|  | EXP* | GCT16 _T1 | 0.222±0.053 | 0.223 (0.189 – 0.240) | 0.938 (0.894 – 0.965) | p=0.175 | N/A | 0.017 – 0.025 | 7.73 – 11.13 |
|  |  | GCT16 _T2 | 0.216±0.054 | 0.202 (0.177 – 0.239) |  |  |  |  |  |
|  |  | GCT16 _T3 | 0.210±0.045 | 0.205 (0.178 – 0.227) |  |  |  |  |  |
|  | UNEXP* | GCT16 _T1 | 0.241±0.053 | 0.246 (0.200 – 0.273) | 0.754 (0.599 – 0.856) |  |  | 0.024 – 0.041 | 9.24 – 13.30 |
|  |  | GCT16 _T2 | 0.240±0.071 | 0.226 (0.207 – 0.249) |  |  |  |  |  |
|  |  | GCT16 _T3 | 0.254±0.122 | 0.236 (0.198 – 0.256) |  |  |  |  |  |
|  | EXP* | RSI16 _T1 | 1.238±0.376 | 1.208 (0.926 – 1.438) | 0.966 (0.943 – 0.980) | p=0.451 | N/A | 0.111 – 0.156 | 9.74 – 13.01 |
|  |  | RSI16 _T2 | 1.265±0.423 | 1.236 (0.896 – 1.571) |  |  |  |  |  |
|  |  | RSI16 _T3 | 1.305±0.377 | 1.321 (1.045 – 1.519) |  |  |  |  |  |
|  | UNEXP* | RSI16 _T1 | 0.700±0.285 | 0.648 (0.482 – 0.855) | 0.918 (0.866 – 0.952) |  |  | 0.125 – 0.171 | 17.40 – 21.86 |
|  |  | RSI16 _T2 | 0.779±0.323 | 0.734 (0.544 – 0.951) |  |  |  |  |  |
|  |  | RSI16 _T3 | 0.794±0.357 | 0.766 (0.534 – 1.017) |  |  |  |  |  |
|  | EXP | JH24 _T1 | 0.283±0.060 | 0.281 (0.248 – 0.320) | 0.970 (0.950 – 0.983) | p=0.865 | N/A | 0.015 – 0.021 | 5.72 – 8.23 |
|  |  | JH24 _T2 | 0.286±0.061 | 0.276 (0.252 – 0.324) |  |  |  |  |  |
|  |  | JH24 _T3 | 0.284±0.062 | 0.282 (0.251 – 0.313) |  |  |  |  |  |
|  | UNEXP | JH24 _T1 | 0.208±0.053 | 0.220 (0.185 – 0.242) | 0.935 (0.894 – 0.962) |  |  | 0.018 – 0.022 | 9.37 – 13.21 |
|  |  | JH24 _T2 | 0.210±0.046 | 0.218 (0.175 – 0.241) |  |  |  |  |  |
|  |  | JH24 _T3 | 0.211±0.050 | 0.221 (0.172 – 0.246) |  |  |  |  |  |
|  | EXP | GCT24 _T1 | 0.214±0.050 | 0.203 (0.178 – 0.240) | 0.970 (0.950 – 0.983) | p=0.720 | N/A | 0.014 – 0.018 | 6.50 – 8.22 |
|  |  | GCT24 _T2 | 0.213±0.046 | 0.206 (0.180 – 0.232) |  |  |  |  |  |
|  |  | GCT24 _T3 | 0.213±0.054 | 0.201 (0.177 – 0.230) |  |  |  |  |  |
|  | UNEXP | GCT24 _T1 | 0.232±0.071 | 0.228 (0.186 – 0.267) | 0.929 (0.884 – 0.958) |  |  | 0.020 – 0.025 | 7.76 – 12.08 |
|  |  | GCT24 _T2 | 0.237±0.058 | 0.239 (0.193 – 0.265) |  |  |  |  |  |
|  |  | GCT24 _T3 | 0.236±0.054 | 0.231 (0.201 – 0.256) |  |  |  |  |  |
|  | EXP | RSI24 _T1 | 1.383±0.413 | 1.370 (1.042 – 1.547) | 0.978 (0.963 – 0.987) | p=0.335 | N/A | 0.111 – 0.126 | 8.47 – 9.59 |
|  |  | RSI24 _T2 | 1.395±0.399 | 1.377 (1.104 – 1.612) |  |  |  |  |  |
|  |  | RSI24 _T3 | 1.393±0.421 | 1.369 (1.114 – 1.616) |  |  |  |  |  |
|  | UNEXP | RSI24 _T1 | 0.973±0.337 | 0.950 (0.748 – 1.184) | 0.952 (0.922 – 0.972) |  |  | 0.096 – 0.123 | 10.05 – 13.11 |
|  |  | RSI24 _T2 | 0.934±0.306 | 0.911 (0.746 – 1.091) |  |  |  |  |  |
|  |  | RSI24 _T3 | 0.959±0.360 | 0.915 (0.722 – 1.123) |  |  |  |  |  |
|  | EXP | JH32 _T1 | 0.293±0.059 | 0.291 (0.269 – 0.331) | 0.967 (0.945 – 0.981) | p=0.520 | N/A | 0.017 – 0.021 | 5.65 – 8.09 |
|  |  | JH32 _T2 | 0.295±0.061 | 0.297 (0.267 – 0.329) |  |  |  |  |  |
|  |  | JH32 _T3 | 0.298±0.070 | 0.293 (0.261 – 0.340) |  |  |  |  |  |
|  | UNEXP | JH32 _T1 | 0.242±0.054 | 0.238 (0.211 – 0.270) | 0.970 (0.951 – 0.983) |  |  | 0.018 | 7.29 – 7.76 |
|  |  | JH32 _T2 | 0.249±0.057 | 0.249 (0.220 – 0.285) |  |  |  |  |  |
|  |  | JH32 _T3 | 0.247±0.058 | 0.250 (0.212 – 0.285) |  |  |  |  |  |
|  | EXP | GCT32 _T1 | 0.210±0.054 | 0.205 (0.171 – 0.227) | 0.945 (0.909 – 0.969) | p=0.733 | N/A | 0.014 – 0.018 | 6.57 – 8.55 |
|  |  | GCT32 _T2 | 0.207±0.042 | 0.202 (0.173 – 0.228) |  |  |  |  |  |
|  |  | GCT32 _T3 | 0.209±0.046 | 0.204 (0.174 – 0.226) |  |  |  |  |  |
|  | UNEXP | GCT32 _T1 | 0.236±0.063 | 0.229 (0.194 – 0.249) | 0.983 (0.972 – 0.990) |  |  | 0.014 – 0.015 | 5.28 – 6.21 |
|  |  | GCT32 _T2 | 0.236±0.070 | 0.228 (0.186 – 0.249) |  |  |  |  |  |
|  |  | GCT32 _T3 | 0.239±0.072 | 0.232 (0.193 – 0.249) |  |  |  |  |  |
|  | EXP | RSI32_T1 | 1.460±0.385 | 1.460 (1.205 – 1.782) | 0.966 (0.943 – 0.981) | p=0.573 | N/A | 0.11 – 0.12 | 7.91 – 9.52 |
|  |  | RSI32 _T2 | 1.469±0.401 | 1.426 (1.188 – 1.710) |  |  |  |  |  |
|  |  | RSI32 _T3 | 1.465±0.406 | 1.433 (1.184 – 1.690) |  |  |  |  |  |
|  | UNEXP | RSI32_T1 | 1.084±0.357 | 1.017 (0.863 – 1.247) | 0.982 (0.971 – 0.990) |  |  | 0.092 – 0.098 | 9.01 – 9.41 |
|  |  | RSI32 _T2 | 1.127±0.394 | 1.112 (0.823 – 1.343) |  |  |  |  |  |
|  |  | RSI32 _T3 | 1.107±0.391 | 1.043 (0.800 – 1.333) |  |  |  |  |  |
| Day 2 | EXP | JH16 _T1 | 0.254±0.058 | 0.263 (0.221 – 0.289) | 0.951 (0.919 – 0.972) | p=0.928 | N/A | 0.019 – 0.026 | 7.88 – 10.76 |
|  |  | JH16 _T2 | 0.259±0.054 | 0.263 (0.222 – 0.304) |  |  |  |  |  |
|  |  | JH16 _T3 | 0.261±0.054 | 0.265 (0.233 – 0.298) |  |  |  |  |  |
|  | UNEXP | JH16 _T1 | 0.184±0.048 | 0.179 (0.151 – 0.221) | 0.967 (0.946 – 0.981) |  |  | 0.013 – 0.018 | 7.48 – 10.76 |
|  |  | JH16 _T2 | 0.189±0.046 | 0.184 (0.155 – 0.210) |  |  |  |  |  |
|  |  | JH16 _T3 | 0.189±0.047 | 0.191 (0.160 – 0.214) |  |  |  |  |  |
|  | EXP | GCT16 _T1 | 0.221±0.049 | 0.218 (0.182 – 0.244) | 0.909 (0.849 – 0.948) | p=0.616 | N/A | 0.015 – 0.027 | 6.63 – 11.47 |
|  |  | GCT16 _T2 | 0.218±0.041 | 0.206 (0.190 – 0.244) |  |  |  |  |  |
|  |  | GCT16 _T3 | 0.215±0.039 | 0.209 (0.182 – 0.243) |  |  |  |  |  |
|  | UNEXP | GCT16 _T1 | 0.237±0.051 | 0.234 (0.220 – 0.253) | 0.928 (0.883 – 0.958) |  |  | 0.014 – 0.020 | 6.07 – 8.06 |
|  |  | GCT16 _T2 | 0.229±0.043 | 0.233 (0.210 – 0.252) |  |  |  |  |  |
|  |  | GCT16 _T3 | 0.226±0.039 | 0.221 (0.208 – 0.249) |  |  |  |  |  |
|  | EXP* | RSI16 _T1 | 1.191±0.327 | 1.216 (0.969 – 1.422) | 0.935 (0.892 – 0.963) | p=0.878 | N/A | 0.119 – 0.179 | 11.10 – 16.53 |
|  |  | RSI16 _T2 | 1.228±0.313 | 1.204 (0.977 – 1.433) |  |  |  |  |  |
|  |  | RSI16 _T3 | 1.244±0.311 | 1.226 (1.072 – 1.424) |  |  |  |  |  |
|  | UNEXP* | RSI16 _T1 | 0.807±0.283 | 0.778 (0.623 – 0.923) | 0.967 (0.946 – 0.980) |  |  | 0.078 – 0.118 | 9.55 – 14.59 |
|  |  | RSI16 _T2 | 0.860±0.309 | 0.766 (0.664 – 1.000) |  |  |  |  |  |
|  |  | RSI16 _T3 | 0.863±0.287 | 0.828 (0.677 – 0.997) |  |  |  |  |  |
|  | EXP* | JH24 _T1 | 0.285±0.056 | 0.287 (0.258 – 0.317) | 0.974 (0.957 – 0.985) | p=0.202 | N/A | 0.015 – 0.016 | 5.35 – 6.36 |
|  |  | JH24 _T2 | 0.281±0.051 | 0.285 (0.256 – 0.319) |  |  |  |  |  |
|  |  | JH24 _T3 | 0.289±0.055 | 0.288 (0.256 – 0.324) |  |  |  |  |  |
|  | UNEXP* | JH24 _T1 | 0.228±0.039 | 0.231 (0.210 – 0.252) | 0.946 (0.913 – 0.969) |  |  | 0.015 – 0.019 | 6.52 – 11.03 |
|  |  | JH24 _T2 | 0.228±0.044 | 0.228 (0.210 – 0.253) |  |  |  |  |  |
|  |  | JH24 _T3 | 0.227±0.050 | 0.236 (0.205 – 0.252) |  |  |  |  |  |
|  | EXP | GCT24 _T1 | 0.207±0.038 | 0.204 (0.182 – 0.224) | 0.947 (0.912 – 0.970) | p=0.556 | N/A | 0.013 – 0.015 | 6.08 – 6.98 |
|  |  | GCT24 _T2 | 0.211±0.038 | 0.207 (0.181 – 0.228) |  |  |  |  |  |
|  |  | GCT24 _T3 | 0.211±0.033 | 0.209 (0.188 – 0.234) |  |  |  |  |  |
|  | UNEXP | GCT24 _T1 | 0.216±0.039 | 0.219 (0.190 – 0.239) | 0.925 (0.878 – 0.956) |  |  | 0.017 – 0.021 | 7.45 – 11.88 |
|  |  | GCT24 _T2 | 0.217±0.037 | 0.221 (0.190 – 0.238) |  |  |  |  |  |
|  |  | GCT24 _T3 | 0.215±0.049 | 0.221 (0.196 – 0.238) |  |  |  |  |  |
|  | EXP* | RSI24 _T1 | 1.405±0.319 | 1.365 (1.148 – 1.620) | 0.978 (0.963 – 0.987) | p=0.430 | N/A | 0.097 – 0.100 | 7.16 – 7.66 |
|  |  | RSI24 _T2 | 1.367±0.329 | 1.325 (1.143 – 1.583) |  |  |  |  |  |
|  |  | RSI24 _T3 | 1.394±0.335 | 1.381 (1.177 – 1.584) |  |  |  |  |  |
|  | UNEXP* | RSI24 _T1 | 1.084±0.283 | 1.035 (0.913 – 1.206) | 0.970 (0.951 – 0.982) |  |  | 0.086 – 0.119 | 8.47 – 11.30 |
|  |  | RSI24 _T2 | 1.080±0.298 | 1.054 (0.947 – 1.210) |  |  |  |  |  |
|  |  | RSI24 _T3 | 1.088±0.323 | 1.048 (0.913 – 1.196) |  |  |  |  |  |
|  | EXP* | JH32 _T1 | 0.298±0.063 | 0.297 (0.256 – 0.334) | 0.967 (0.945 – 0.981) | p=0.668 | N/A | 0.015 – 0.019 | 5.30 – 6.70 |
|  |  | JH32 _T2 | 0.301±0.064 | 0.307 (0.265 – 0.349) |  |  |  |  |  |
|  |  | JH32 _T3 | 0.299±0.061 | 0.194 (0.263 – 0.342) |  |  |  |  |  |
|  | UNEXP* | JH32 _T1 | 0.253±0.055 | 0.255 (0.213 – 0.294) | 0.975 (0.959 – 0.985) |  |  | 0.017 – 0.019 | 6.81 – 7.00 |
|  |  | JH32 _T2 | 0.260±0.060 | 0.254 (0.223 – 0.301) |  |  |  |  |  |
|  |  | JH32 _T3 | 0.258±0.061 | 0.243 (0.222 – 0.316) |  |  |  |  |  |
|  | EXP* | GCT32 _T1 | 0.204±0.034 | 0.202 (0.179 – 0.224) | 0.946 (0.909 – 0.969) | p=0.994 | N/A | 0.011 – 0.015 | 5.33 – 7.23 |
|  |  | GCT32 _T2 | 0.207±0.031 | 0.205 (0.184 – 0.224) |  |  |  |  |  |
|  |  | GCT32 _T3 | 0.205±0.032 | 0.206 (0.179 – 0.226) |  |  |  |  |  |
|  | UNEXP* | GCT32 _T1 | 0.219±0.043 | 0.213 (0.195 – 0.245) | 0.960 (0.935 – 0.977) |  |  | 0.015 – 0.016 | 7.00 – 7.35 |
|  |  | GCT32 _T2 | 0.222±0.042 | 0.218 (0.199 – 0.241) |  |  |  |  |  |
|  |  | GCT32 _T3 | 0.220±0.039 | 0.218 (0.198 – 0.237) |  |  |  |  |  |
|  | EXP* | RSI32_T1 | 1.487±0.354 | 1.481 (1.162 – 1.728) | 0.966 (0.943 – 0.981) | P=0.676 | N/A | 0.104 – 0.130 | 7.55 – 9.25 |
|  |  | RSI32 _T2 | 1.484±0.372 | 1.506 (1.258 – 1.725) |  |  |  |  |  |
|  |  | RSI32 _T3 | 1.489±0.366 | 1.534 (1.183 – 1.747) |  |  |  |  |  |
|  | UNEXP* | RSI32_T1 | 1.182±0.299 | 1.187 (0.943 – 1.365) | 0.957 (0.930 – 0.975) |  |  | 0.115 – 0.134 | 9.68 – 11.44 |
|  |  | RSI32 _T2 | 1.207±0.349 | 1.208 (0.944 – 1.417) |  |  |  |  |  |
|  |  | RSI32 _T3 | 1.204±0.355 | 1.137 (0.891 – 1.454) |  |  |  |  |  |
| Day 3 | EXP* | JH16 _T1 | 0.259±0.053 | 0.272 (0.230 – 0.296) | 0.949 (0.915 – 0.971) | p=0.224 | N/A | 0.020 – 0.025 | 8.05 – 10.41 |
|  |  | JH16 _T2 | 0.260±0.059 | 0.263 (0.209 – 0.307) |  |  |  |  |  |
|  |  | JH16 _T3 | 0.270±0.061 | 0.275 (0.229 – 0.306) |  |  |  |  |  |
|  | UNEXP* | JH16 _T1 | 0.192±0.040 | 0.188 (0.165 – 0.213) | 0.937 (0.898 – 0.963) |  |  | 0.014 – 0.019 | 7.34 – 9.65 |
|  |  | JH16 _T2 | 0.196±0.044 | 0.192 (0.168 – 0.226) |  |  |  |  |  |
|  |  | JH16 _T3 | 0.195±0.044 | 0.193 (0.166 – 0.226) |  |  |  |  |  |
|  | EXP | GCT16 _T1 | 0.212±0.041 | 0.201 (0.181 – 0.239) | 0.943 (0.905 – 0.968) | ηp²=0.044  p=0.020 | UNEXP  T1_T3: p<0.001, d=0.41 | 0.014 – 0.018 | 6.65 – 8.09 |
|  |  | GCT16 _T2 | 0.212±0.036 | 0.208 (0.179 – 0.239) |  |  |  |  |  |
|  |  | GCT16 _T3 | 0.211±0.036 | 0.216 (0.180 – 0.232) |  |  |  |  |  |
|  | UNEXP | GCT16 _T1 | 0.225±0.036 | 0.224 (0.199 – 0.245) | 0.909 (0.852 – 0.947) |  |  | 0.015 – 0.023 | 6.38 – 10.28 |
|  |  | GCT16 _T2 | 0.219±0.036 | 0.216 (0.200 – 0.241) |  |  |  |  |  |
|  |  | GCT16 _T3 | 0.210±0.030 | 0.210 (0.191 – 0.230) |  |  |  |  |  |
|  | EXP* | RSI16 _T1 | 1.259±0.334 | 1.234 (0.966 – 1.475) | 0.952 (0.921 – 0.973) | p=0.558 | N/A | 0.117 – 0.156 | 9.81 – 12.91 |
|  |  | RSI16 _T2 | 1.263±0.365 | 1.208 (0.948 – 1.557) |  |  |  |  |  |
|  |  | RSI16 _T3 | 1.306±0.343 | 1.293 (1.080 – 1.495) |  |  |  |  |  |
|  | UNEXP* | RSI16 _T1 | 0.879±0.266 | 0.787 (0.694 – 1.031) | 0.957 (0.929 – 0.974) |  |  | 0.099 – 0.120 | 11.13 – 12.87 |
|  |  | RSI16 _T2 | 0.919±0.285 | 0.889 (0.719 – 1.034) |  |  |  |  |  |
|  |  | RSI16 _T3 | 0.951±0.290 | 0.894 (0.764 – 1.063) |  |  |  |  |  |
|  | EXP* | JH24 _T1 | 0.283±0.054 | 0.287 (0.247 – 0.324) | 0.967 (0.946 – 0.981) | p=0424 | N/A | 0.014 – 0.018 | 5.50 – 5.97 |
|  |  | JH24 _T2 | 0.285±0.049 | 0.296 (0.255 – 0.322) |  |  |  |  |  |
|  |  | JH24 _T3 | 0.286±0.052 | 0.287 (0.258 – 0.318) |  |  |  |  |  |
|  | UNEXP* | JH24 _T1 | 0.225±0.040 | 0.237 (0.208 – 0.250) | 0.957 (0.929 – 0.975) |  |  | 0.014 – 0.018 | 6.98 – 8.06 |
|  |  | JH24 _T2 | 0.228±0.044 | 0.232 (0.205 – 0.254) |  |  |  |  |  |
|  |  | JH24 _T3 | 0.234±0.042 | 0.241 (0.208 – 0.260) |  |  |  |  |  |
|  | EXP | GCT24 _T1 | 0.210±0.040 | 0.206 (0.183 – 0.238) | 0.938 (0.896 – 0.965) | p=0.926 | N/A | 0.015 – 0.018 | 6.89 – 8.16 |
|  |  | GCT24 _T2 | 0.209±0.035 | 0.201 (0.180 – 0.234) |  |  |  |  |  |
|  |  | GCT24 _T3 | 0.208±0.039 | 0.204 (0.177 – 0.227) |  |  |  |  |  |
|  | UNEXP | GCT24 _T1 | 0.210±0.040 | 0.217 (0.196 – 0.231) | 0.950 (0.918 – 0.970) |  |  | 0.012 – 0.013 | 5.91 – 6.39 |
|  |  | GCT24 _T2 | 0.211±0.028 | 0.211 (0.191 – 0.236) |  |  |  |  |  |
|  |  | GCT24 _T3 | 0.212±0.034 | 0.213 (0.188 – 0.236) |  |  |  |  |  |
|  | EXP* | RSI24 _T1 | 1.383±0.344 | 1.300 (1.114 – 1.610) | 0.962 (0.936 – 0.978) | p=0.749 | N/A | 0.114 – 0.122 | 8.56 – 9.35 |
|  |  | RSI24 _T2 | 1.391±0.315 | 1.364 (1.131 – 1.679) |  |  |  |  |  |
|  |  | RSI24 _T3 | 1.406±0.329 | 1.354 (1.131 – 1.594) |  |  |  |  |  |
|  | UNEXP* | RSI24 _T1 | 1.081±0.253 | 1.060 (0.913 – 1.224) | 0.956 (0.928 – 0.974) |  |  | 0.098 – 0.105 | 9.13 – 9.94 |
|  |  | RSI24 _T2 | 1.097±0.278 | 1.077 (0.913 – 1.201) |  |  |  |  |  |
|  |  | RSI24 _T3 | 1.127±0.268 | 1.117 (0.953 – 1.214) |  |  |  |  |  |
|  | EXP* | JH32 _T1 | 0.300±0.057 | 0.298 (0.260 – 0.337) | 0.963 (0.939 – 0.979) | p=0.604 | N/A | 0.015 – 0.020 | 5.66 – 7.04 |
|  |  | JH32 _T2 | 0.300±0.060 | 0.300 (0.257 – 0.345) |  |  |  |  |  |
|  |  | JH32 _T3 | 0.301±0.059 | 0.303 (0.258 – 0.347) |  |  |  |  |  |
|  | UNEXP* | JH32 _T1 | 0.262±0.061 | 0.258 (0.215 – 0.315) | 0.983 (0.971 – 0.990) |  |  | 0.015 – 0.016 | 6.07 – 6.60 |
|  |  | JH32 _T2 | 0.266±0.062 | 0.268 (0.216 – 0.314) |  |  |  |  |  |
|  |  | JH32 _T3 | 0.267±0.064 | 0.271 (0.207 – 0.321) |  |  |  |  |  |
|  | EXP | GCT32 _T1 | 0.203±0.037 | 0.196 (0.176 – 0.223) | 0.946 (0.910 – 0.969) | p=0.410 | N/A | 0.011 – 0.016 | 6.86 – 7.83 |
|  |  | GCT32 _T2 | 0.200±0.036 | 0.198 (0.174 – 0.218) |  |  |  |  |  |
|  |  | GCT32 _T3 | 0.201±0.033 | 0.202 (0.176 – 0.218) |  |  |  |  |  |
|  | UNEXP | GCT32 _T1 | 0.210±0.032 | 0.210 (0.193 – 0.232) | 0.950 (0.918 – 0.970) |  |  | 0.013 – 0.015 | 6.05 – 6.94 |
|  |  | GCT32 _T2 | 0.213±0.035 | 0.210 (0.193 – 0.240) |  |  |  |  |  |
|  |  | GCT32 _T3 | 0.211±0.034 | 0.209 (0.188 – 0.236) |  |  |  |  |  |
|  | EXP* | RSI32_T1 | 1.516±0.360 | 1.486 (1.206 – 1.755) | 0.963 (0.938 – 0.979) | p=0.823 | N/A | 0.107 – 0.132 | 7.58 – 9.93 |
|  |  | RSI32 _T2 | 1.527±0.358 | 1.523 (1.226 – 1.812) |  |  |  |  |  |
|  |  | RSI32 _T3 | 1.528±0.359 | 1.484 (1.219 – 1.822) |  |  |  |  |  |
|  | UNEXP* | RSI32_T1 | 1.266±0.320 | 1.235 (1.011 – 1.523) | 0.978 (0.956 – 0.984) |  |  | 0.098 – 0.113 | 8.01 – 8.93 |
|  |  | RSI32 _T2 | 1.281±0.357 | 1.248 (1.007 – 1.522) |  |  |  |  |  |
|  |  | RSI32 _T3 | 1.297±0.379 | 1.265 (1.046 – 1.532) |  |  |  |  |  |
| Day 4 | EXP* | JH16 _T1 | 0.252±0.058 | 0.253 (0.213 – 0.290) | 0.911 (0.851 – 0.949) | p=0.823 | N/A | 0.022 – 0.030 | 11.40 – 17.37 |
|  |  | JH16 _T2 | 0.253±0.070 | 0.251 (0.214 – 0.303) |  |  |  |  |  |
|  |  | JH16 _T3 | 0.250±0.075 | 0.258 (0.216 – 0.298) |  |  |  |  |  |
|  | UNEXP* | JH16 _T1 | 0.196±0.039 | 0.192 (0.164 – 0.217) | 0.898 (0.834 – 0.940) |  |  | 0.015 – 0.020 | 7.80 – 12.49 |
|  |  | JH16 _T2 | 0.199±0.043 | 0.193 (0.168 – 0.223) |  |  |  |  |  |
|  |  | JH16 _T3 | 0.196±0.051 | 0.194 (0.168 – 0.223) |  |  |  |  |  |
|  | EXP | GCT16 _T1 | 0.210±0.039 | 0.204 (0.182 – 0.236) | 0.765 (0.608 – 0.866) | p=0.706 | N/A | 0.021 – 0.029 | 10.87 – 16.35 |
|  |  | GCT16 _T2 | 0.207±0.049 | 0.210 (0.176 – 0.246) |  |  |  |  |  |
|  |  | GCT16 _T3 | 0.208±0.054 | 0.203 (0.176 – 0.249) |  |  |  |  |  |
|  | UNEXP | GCT16 _T1 | 0.215±0.035 | 0.213 (0.191 – 0.241) | 0.907 (0.848 – 0.945) |  |  | 0.012 – 0.018 | 5.69 – 9.41 |
|  |  | GCT16 _T2 | 0.210±0.039 | 0.210 (0.189 – 0.236) |  |  |  |  |  |
|  |  | GCT16 _T3 | 0.207±0.041 | 0.208 (0.185 – 0.233) |  |  |  |  |  |
|  | EXP* | RSI16 _T1 | 1.240±0.381 | 1.160 (0.984 – 1.470) | 0.699 (0.498 – 0.828) | p =0.529 | N/A | 0.125 – 0.248 | 13.28 – 16.83 |
|  |  | RSI16 _T2 | 1.233±0.393 | 1.194 (0.980 – 1.483) |  |  |  |  |  |
|  |  | RSI16 _T3 | 1.321±0.678 | 1.181 (0.986 – 1.462) |  |  |  |  |  |
|  | UNEXP* | RSI16 _T1 | 0.937±0.240 | 0.894 (0.768 – 1.065) | 0.923 (0.875 – 0.955) |  |  | 0.09 – 0.129 | 10.56 – 15.67 |
|  |  | RSI16 _T2 | 0.966±0.259 | 0.926 (0.800 – 1.110) |  |  |  |  |  |
|  |  | RSI16 _T3 | 0.961±0.326 | 0.895 (0.779 – 1.067) |  |  |  |  |  |
|  | EXP* | JH24 _T1 | 0.274±0.074 | 0.288 (0.238 – 0.313) | 0.923 (0.871 – 0.956) | p =0.988 | N/A | 0.018 – 0.025 | 6.72 – 11.62 |
|  |  | JH24 _T2 | 0.284±0.054 | 0.277 (0.246 – 0.311) |  |  |  |  |  |
|  |  | JH24 _T3 | 0.281±0.060 | 0.281 (0.257 – 0.305) |  |  |  |  |  |
|  | UNEXP* | JH24 _T1 | 0.227±0.059 | 0.240 (0.207 – 0.255) | 0.885 (0.813 – 0.933) |  |  | 0.015 – 0.023 | 6.49 – 14.45 |
|  |  | JH24 _T2 | 0.236±0.041 | 0.243 (0.204 – 0.257) |  |  |  |  |  |
|  |  | JH24 _T3 | 0.235±0.044 | 0.241 (0.202 – 0.246) |  |  |  |  |  |
|  | EXP | GCT24 _T1 | 0.208±0.049 | 0.205 (0.182 – 0.239) | 0.827 (0.711 – 0.901) | p =0.988 | N/A | 0.015 – 0.023 | 7.20 – 12.33 |
|  |  | GCT24 _T2 | 0.207±0.030 | 0.198 (0.175 – 0.233) |  |  |  |  |  |
|  |  | GCT24 _T3 | 0.209±0.033 | 0.199 (0.184 – 0.229) |  |  |  |  |  |
|  | UNEX | GCT24 _T1 | 0.201±0.052 | 0.209 (0.182 – 0.223) | 0.716 (0.536 – 0.833) |  |  | 0.012 – 0.020 | 5.94 – 12.56 |
|  |  | GCT24 _T2 | 0.207±0.030 | 0.206 (0.189 – 0.231) |  |  |  |  |  |
|  |  | GCT24 _T3 | 0.209±0.033 | 0.206 (0.189 – 0.229) |  |  |  |  |  |
|  | EXP* | RSI24 _T1 | 1.338±0.375 | 1.342 (1.075 – 1.555) | 0.953 (0.921 – 0.973) | p=0.288 | N/A | 0.133 – 0.155 | 10.08 – 12.09 |
|  |  | RSI24 _T2 | 1.421±0.364 | 1.379 (1.108 – 1.608) |  |  |  |  |  |
|  |  | RSI24 _T3 | 1.413±0.378 | 1.407 (1.140 – 1.572) |  |  |  |  |  |
|  | UNEXP* | RSI24 _T1 | 1.136±0.321 | 1.158 (0.954 – 1.285) | 0.956 (0.929 – 0.974) |  |  | 0.100 – 0.112 | 8.95 – 12.46 |
|  |  | RSI24 _T2 | 1.172±0.307 | 1.169 (0.923 – 1.315) |  |  |  |  |  |
|  |  | RSI24 _T3 | 1.149±0.313 | 1.127 (0.951 – 1.327) |  |  |  |  |  |
|  | EXP | JH32 _T1 | 0.292±0.062 | 0.276 (0.262 – 0.334) | 0.986 (0.977 – 0.992) | p=0.355 | N/A | 0.013 – 0.015 | 4.79 – 5.52 |
|  |  | JH32 _T2 | 0.295±0.068 | 0.288 (0.257 – 0.339) |  |  |  |  |  |
|  |  | JH32 _T3 | 0.295±0.065 | 0.290 (0.257 – 0.333) |  |  |  |  |  |
|  | UNEXP | JH32 _T1 | 0.269±0.067 | 0.266 (0.204 – 0.330) | 0.985 (0.976 – 0.991) |  |  | 0.014 – 0.018 | 5.63 – 6.92 |
|  |  | JH32 _T2 | 0.268±0.069 | 0.270 (0.204 – 0.329) |  |  |  |  |  |
|  |  | JH32 _T3 | 0.273±0.069 | 0.265 (0.215 – 0.340) |  |  |  |  |  |
|  | EXP | GCT32 _T1 | 0.203±0.033 | 0.205 (0.185 – 0.227) | 0.933 (0.888 – 0.962) | p=0.722 | N/A | 0.014 – 0.016 | 6.78 – 7.34 |
|  |  | GCT32 _T2 | 0.202±0.031 | 0.199 (0.176 – 0.227) |  |  |  |  |  |
|  |  | GCT32 _T3 | 0.204±0.029 | 0.200 (0.176 – 0.224) |  |  |  |  |  |
|  | UNEXP | GCT32 _T1 | 0.205±0.029 | 0.205 (0.185 – 0.227) | 0.959 (0.934 – 0.976) |  |  | 0.010 – 0.015 | 4.82 – 5.84 |
|  |  | GCT32 _T2 | 0.207±0.031 | 0.205 (0.185 – 0.229) |  |  |  |  |  |
|  |  | GCT32 _T3 | 0.204±0.032 | 0.204 (0.187 – 0.229) |  |  |  |  |  |
|  | EXP | RSI32_T1 | 1.483±0.418 | 1.409 (1.169 – 1.778) | 0.981 (0.968 – 0.989) | p=0.444 | N/A | 0.108 – 0.112 | 7.63 – 8.24 |
|  |  | RSI32 _T2 | 1.491±0.406 | 1.434 (1.149 – 1.734) |  |  |  |  |  |
|  |  | RSI32 _T3 | 1.503±0.421 | 1.467 (1.184 – 1.836) |  |  |  |  |  |
|  | UNEXP | RSI32_T1 | 1.344±0.398 | 1.290 (1.000 – 1.685) | 0.984 (0.973 – 0.990) |  |  | 0.088 – 0.111 | 6.94 – 8.72 |
|  |  | RSI32 _T2 | 1.324±0.395 | 1.294 (0.938 – 1.615) |  |  |  |  |  |
|  |  | RSI32 _T3 | 1.369±0.424 | 1.324 (1.018 – 1.720) |  |  |  |  |  |
| Day 5 | EXP* | JH16 _T1 | 0.256±0.063 | 0.264 (0.204 – 0.295) | 0.985 (0.975 – 0.992) | p=0.697 | N/A | 0.014 – 0.016 | 6.01 – 7.74 |
|  |  | JH16 _T2 | 0.259±0.069 | 0.262 (0.202 – 0.312) |  |  |  |  |  |
|  |  | JH16 _T3 | 0.266±0.066 | 0.264 (0.212 – 0.326) |  |  |  |  |  |
|  | UNEXP* | JH16 _T1 | 0.190±0.045 | 0.179 (0.167 – 0.215) | 0.964 (0.942 – 0.979) |  |  | 0.013 – 0.017 | 6.98 – 9.20 |
|  |  | JH16 _T2 | 0.196±0.044 | 0.193 (0.168 – 0.228) |  |  |  |  |  |
|  |  | JH16 _T3 | 0.199±0.066 | 0.191 (0.175 – 0.224) |  |  |  |  |  |
|  | EXP | GCT16 _T1 | 0.204±0.032 | 0.200 (0.182 – 0.222) | 0.888 (0.814 – 0.936) | p=0.382 | N/A | 0.012 – 0.020 | 5.73 – 9.41 |
|  |  | GCT16 _T2 | 0.206±0.033 | 0.203 (0.179 – 0.225) |  |  |  |  |  |
|  |  | GCT16 _T3 | 0.201±0.032 | 0.198 (0.178 – 0.218) |  |  |  |  |  |
|  | UNEXP | GCT16 _T1 | 0.214±0.035 | 0.210 (0.196 – 0.235) | 0.944 (0.909 – 0.967) |  |  | 0.012 – 0.016 | 5.86 – 7.71 |
|  |  | GCT16 _T2 | 0.210±0.035 | 0.208 (0.189 – 0.229) |  |  |  |  |  |
|  |  | GCT16 _T3 | 0.206±0.033 | 0.206 (0.183 – 0.229) |  |  |  |  |  |
|  | EXP* | RSI16 _T1 | 1.286±0.383 | 1.321 (0.941 – 1.520) | 0.972 (0.954 – 0.984) | p=0.233 | N/A | 0.111 – 0.146 | 9.00 – 12.17 |
|  |  | RSI16 _T2 | 1.282±0.383 | 1.321 (1.030 – 1.522) |  |  |  |  |  |
|  |  | RSI16 _T3 | 1.349±0.396 | 1.324 (1.043 – 1.575) |  |  |  |  |  |
|  | UNEXP* | RSI16 _T1 | 0.915±0.292 | 0.845 (0.715 – 1.039) | 0.966 (0.944 – 0.980) |  |  | 0.107 – 0.113 | 11.49 – 12.69 |
|  |  | RSI16 _T2 | 0.961±0.299 | 0.919 (0.795 – 1.060) |  |  |  |  |  |
|  |  | RSI16 _T3 | 0.991±0.295 | 0.924 (0.817 – 1.101) |  |  |  |  |  |
|  | EXP* | JH24 _T1 | 0.283±0.064 | 0.286 (0.251 – 0.332) | 0.978 (0.964 – 0.988) | p=0.998 | N/A | 0.013 – 0.018 | 5.95 – 6.14 |
|  |  | JH24 _T2 | 0.286±0.066 | 0.286 (0.245 – 0.331) |  |  |  |  |  |
|  |  | JH24 _T3 | 0.287±0.065 | 0.283 (0.253 – 0.332) |  |  |  |  |  |
|  | UNEXP* | JH24 _T1 | 0.234±0.045 | 0.238 (0.210 – 0.269) | 0.964 (0.941 – 0.979) |  |  | 0.013 – 0.017 | 5.60 – 6.14 |
|  |  | JH24 _T2 | 0.237±0.042 | 0.244 (0.205 – 0.270) |  |  |  |  |  |
|  |  | JH24 _T3 | 0.239±0.046 | 0.246 (0.202 – 0.275) |  |  |  |  |  |
|  | EXP | GCT24 _T1 | 0.206±0.034 | 0.204 (0.176 – 0.223) | 0.919 (0.865 – 0.954) | p=0.879 | N/A | 0.015 – 0.019 | 7.16 – 8.65 |
|  |  | GCT24 _T2 | 0.206±0.039 | 0.204 (0.176 – 0.229) |  |  |  |  |  |
|  |  | GCT24 _T3 | 0.201±0.038 | 0.198 (0.178 – 0.213) |  |  |  |  |  |
|  | UNEXP | GCT24 _T1 | 0.207±0.034 | 0.201 (0.184 – 0.232) | 0.959 (0.934 – 0.976) |  |  | 0.011 – 0.013 | 5.44 – 5.94 |
|  |  | GCT24 _T2 | 0.206±0.033 | 0.205 (0.185 – 0.228) |  |  |  |  |  |
|  |  | GCT24 _T3 | 0.204±0.033 | 0.202 (0.183 – 0.226) |  |  |  |  |  |
|  | EXP* | RSI24 _T1 | 1.413±0.406 | 1.365 (1.205 – 1.655) | 0.978 (0.963 – 0.987) | p=0.909 | N/A | 0.106 – 0.118 | 7.79 – 10.03 |
|  |  | RSI24 _T2 | 1.432±0.420 | 1.434 (1.176 – 1.628) |  |  |  |  |  |
|  |  | RSI24 _T3 | 1.469±0.403 | 1.496 (1.195 – 1.722) |  |  |  |  |  |
|  | UNEXP* | RSI24 _T1 | 1.163±0.313 | 1.134 (0.942 – 1.361) | 0.973 (0.956 – 0.984) |  |  | 0.088 – 0.111 | 8.057 – 9.946 |
|  |  | RSI24 _T2 | 1.186±0.312 | 1.093 (1.014 – 1.398) |  |  |  |  |  |
|  |  | RSI24 _T3 | 1.211±0.336 | 1.179 (0.983 – 1.455) |  |  |  |  |  |
|  | EXP* | JH32 _T1 | 0.300±0.074 | 0.298 (0.254 – 0.341) | 0.981 (0.969 – 0.989) | p=0.888 | N/A | 0.011 – 0.021 | 3.93 – 6.82 |
|  |  | JH32 _T2 | 0.299±0.067 | 0.293 (0.252 – 0.350) |  |  |  |  |  |
|  |  | JH32 _T3 | 0.302±0.070 | 0.291 (0.255 – 0.353) |  |  |  |  |  |
|  | UNEXP* | JH32 _T1 | 0.269±0.073 | 0.267 (0.206 – 0.324) | 0.977 (0.963 – 0.987) |  |  | 0.017 | 6.40 – 8.47 |
|  |  | JH32 _T2 | 0.268±0.070 | 0.263 (0.211 – 0.325) |  |  |  |  |  |
|  |  | JH32 _T3 | 0.273±0.010 | 0.259 (0.217 – 0.334) |  |  |  |  |  |
|  | EXP* | GCT32 _T1 | 0.206±0.032 | 0.205 (0.181 – 0.223) | 0.899 (0.831 – 0.942) | p=0.820 | N/A | 0.017 – 0.019 | 7.57 – 8.66 |
|  |  | GCT32 _T2 | 0.204±0.030 | 0.202 (0.181 – 0.226) |  |  |  |  |  |
|  |  | GCT32 _T3 | 0.210±0.030 | 0.207 (0.186 – 0.234) |  |  |  |  |  |
|  | UNEXP* | GCT32 _T1 | 0.208±0.031 | 0.202 (0.190 – 0.231) | 0.951 (0.919 – 0.971) |  |  | 0.010 – 0.015 | 5.02 – 7.19 |
|  |  | GCT32 _T2 | 0.207±0.031 | 0.204 (0.191 – 0.222) |  |  |  |  |  |
|  |  | GCT32 _T3 | 0.210±0.036 | 0.214 (0.184 – 0.233) |  |  |  |  |  |
|  | EXP | RSI32_T1 | 1.492±0.448 | 1.446 (1.163 – 1.787) | 0.972 (0.954 – 0.984) | p=0.984 | N/A | 0.110 – 0.158 | 8.12 – 11.00 |
|  |  | RSI32 _T2 | 1.499±0.422 | 1.510 (1.134 – 1.714) |  |  |  |  |  |
|  |  | RSI32 _T3 | 1.475±0.413 | 1.509 (1.147 – 1.759) |  |  |  |  |  |
|  | UNEXP | RSI32_T1 | 1.328±0.443 | 1.327 (1.004 – 1.644) | 0.967 (0.946 – 0.981) |  |  | 0.105 – 0.149 | 9.92 – 11.19 |
|  |  | RSI32 _T2 | 1.333±0.417 | 1.261 (1.000 – 1.656) |  |  |  |  |  |
|  |  | RSI32 _T3 | 1.356±0.463 | 1.270 (1.024 – 1.689) |  |  |  |  |  |

*Legend: EXP = experienced participants; UNEXP = inexperienced participants; JH = jump height; GCT = ground contact time; RSI = reactive strength index; M = mean; SD = standard deviation;ICC = intraclass correlation coefficient; SEM = standard error of measurement; MDC = minimal detectable change; MAE = mean absolute error; MAPE = mean absolute percentage error; T1-T5 = testing days 1 to 5*
